# Rapid chemical de-N-glycosylation and derivatization for liquid chromatography of immunoglobulin N-linked glycans

**DOI:** 10.1371/journal.pone.0196800

**Published:** 2018-05-03

**Authors:** Akihiko Kameyama, Santha Kumara Dissanayake, Wai Wai Thet Tin

**Affiliations:** Biotechnology Research Institute for Drug Discovery, National Institute of Advanced Industrial Science and Technology (AIST), Tsukuba, Ibaraki, Japan; University of Insubria, ITALY

## Abstract

Glycan analysis may result in exploitation of glycan biomarkers and evaluation of heterogeneity of glycosylation of biopharmaceuticals. For N-linked glycan analysis, we investigated alkaline hydrolysis of the asparagine glycosyl carboxamide of glycoproteins as a deglycosylation reaction. By adding hydroxylamine into alkaline de-N-glycosylation, we suppressed the degradation of released glycans and obtained a mixture of oximes, free glycans, and glycosylamines. The reaction was completed within 1 h, and the mixture containing oximes was easily tagged with 2-aminobenzamide by reductive amination. Here, we demonstrated N-linked glycan analysis using this method for a monoclonal antibody, and examined whether this method could liberate glycans without degradation from apo-transferrin containing NeuAc and NeuGc and horseradish peroxidase containing Fuc α1–3 GlcNAc at the reducing end. Furthermore, we compared glycan recoveries between conventional enzymatic glycan release and this method. Increasing the reaction temperature and reaction duration led to degradation, whereas decreasing these parameters resulted in lower release. Considering this balance, we proposed to carry out the reaction at 80°C for 1 h for asialo glycoproteins from mammals and at 50°C for 1 h for sialoglycoproteins.

## Introduction

Protein glycosylation is the most common post-translational modification of proteins and regulates the function, stability, and solubility of glycosylated proteins. Glycans are assembled on proteins by a suite of glycosyltransferases on the Golgi apparatus and endoplasmic reticulum within a cell. These glycosyltransferases vary depending on the lineage and differentiation stage of cells, tissues, and species. Recently, glycan analysis of biopharmaceutical proteins has attracted attention. Therapeutic monoclonal antibodies, which now represent the most rapidly expanding category of ethical pharmaceuticals, normally have N-linked glycans at their Fc region. These glycans are involved in the safety and efficacy mechanisms of therapeutic antibodies, including antibody-dependent cell-mediated cytotoxicity (ADCC) [1], complement-dependent cytotoxicity (CDC) [2,3], and clearance rate [4]. The glycan profile of therapeutic antibodies is influenced by the host cell line and cell culture conditions used for the production and manufacturing process [5]. To maintain the consistency of glycosylation in the production of therapeutics, knowledge of the glycan profile for each step and at any given time during the culture period is desired. In this context, a simple rapid method for glycan analysis is urgently needed.

Glycans are usually subjected to derivatization, such as permethylation and reductive amination, into an appropriate form for analytical methods, such as mass spectrometry [6], high-performance liquid chromatography (HPLC) [7,8], and capillary electrophoresis, prior to analysis [9]. Glycan preparation, however, requires burdensome pretreatment of glycoproteins. To obtain N-linked glycans, denaturation, protease digestion, peptide-N-glycosidase digestion, and purification of glycans are usually performed. This procedure often takes 24 h and requires expensive enzymes, such as PNGase F or A [10]. Some improvements to the deglycosylation method using PNGase F have been reported using microwave-assisted reactions [11], pressure-cycling technology [12], and immobilization of PNGase F [13,14]. These methods can reduce the reaction duration and amount of expensive enzyme needed, but still require special equipment or devices.

Chemical deglycosylation using hydrazine has also been used to prepare N-linked glycans from glycoproteins. In contrast to PNGase F digestion, intact proteins can be subjected to hydrazinolysis without denaturation and protease digestion. However, this reaction requires completely anhydrous conditions and takes a long time for preparation; moreover, anhydrous hydrazine must be handled with meticulous care because it is extremely toxic and hazardous [15,16]. Furthermore, glycans containing N-glycolylneuraminic acid (NeuGc) cannot be obtained by this method because all the amide groups are decomposed and then reacetylated [17]. Because NeuGc is a nonhuman structure, its presence or absence in therapeutic antibodies is an important issue [18]. Therefore, hydrazinolysis is an unsuitable method for glycan profiling of biopharmaceutical proteins. Reductive beta elimination using a dilute base in the presence of sodium borohydride has often been used to release O-linked glycans from glycoproteins. N-linked glycans are also detected as minor products in the glycan mixture obtained from this reaction [19,20]. O-linked glycans are reduced into the corresponding alditols in the reaction, whereas N-linked glycans are detected in their unreduced form. Huang et al. have reported that ammonia-based nonreductive beta elimination releases N-linked glycans as glycosylamines that can be easily hydrolyzed to reducing glycans [21], suggesting that the N-linked glycan release in these reactions may be related to mild alkaline hydrolysis of the glycosyl carboxamide of Asn. In contrast to hydrazinolysis, ammonia-based beta elimination does not decompose amide groups and can also afford NeuGc containing glycans. The reaction, however, takes 40 h, which should still be further improved [21].

Glycans are unstable under basic conditions. Therefore, the glycans released by alkaline hydrolysis of the glycosyl carboxamide of Asn decompose during the reaction. Hydroxylamine reacts with the reducing end of a glycan to give the corresponding oxime, which is more stable than the reducing glycan under basic conditions. In the presence of hydroxylamine, chemical deglycosylation of N-linked glycans using alkaline conditions may improve the yield of the released glycans. Here, we examined the reaction and found that N-linked glycans were released within 1 h at 50°C or 80°C. The released glycans and the corresponding oximes were labeled with a fluorescent tag (2-aminobenzamide [2-AB]) that enabled their sensitive analysis by HPLC. Furthermore, we demonstrated the chemical de-N-glycosylation of some model glycoproteins, including a monoclonal antibody, bovine apo-transferrin, and horseradish peroxidase (HRP), and discussed the advantages of the method.

## Materials and methods

### Materials and reagents

The monoclonal antibody MAB-1-L001 (IgG_1_), produced in CHO cells, was provided by the Manufacturing Technology Association of Biologics (Tokyo, Japan). Hydroxylamine solution (50 wt.% in water), lithium hydroxide monohydrate, and bovine apo-transferrin were purchased from Sigma-Aldrich (St. Louis, MO, USA). PNGase F and glycopeptidase A (from Almond) were purchased from Takara Bio Inc. (Otsu, Japan) and Seikagaku Biobusiness Corp. (Tokyo, Japan), respectively. 2-Picoline borane and 2-aminobenzamide were purchased from Junsei Chemical Co., Ltd. (Tokyo, Japan) and Tokyo Chemical Industry Co., Ltd. (Tokyo, Japan), respectively. HRP and other chemicals and solvents were purchased from Wako Pure Chemical (Osaka, Japan).

### Chemical de-N-glycosylation and purification

Hydroxylamine solution (5–20 μL) and 10 μL of 0.5 M lithium hydroxide were added to 20 μL glycoprotein solution (1–10 mg/mL). The mixture was heated at 50°C or 80°C for the specified period. After cooling to room temperature, the mixture was neutralized with 1 N hydrochloric acid in an ice bath, diluted to 1 mL with water, and applied to a HyperSep Hypercarb SPE cartridge (25 mg; Thermo Fisher Scientific Inc., TN, USA). After washing with water (1 mL) and then with 0.1% TFA (1 mL), glycans and oximes were eluted with 1 mL of 50% acetonitrile containing 0.1% TFA. The eluted solution was dried *in vacuo* and then used as the starting material for the 2-AB labeling procedure.

### Enzymatic deglycosylation

Glycan liberation using PNGase F or glycopeptidase A was performed according to the manufacturer’s instructions. For PNGase F digestion, proteins (40–200 μg) were dissolved in 40 μL of 500 mM Tris-HCl buffer (pH 8.6) containing 50 mM 2-mercaptoethanol and 0.1% sodium dodecyl sulfate. The mixture was heated to 80°C and incubated for 10 min. After cooling to room temperature, 40 μL of 1% Nonidet P-40, 15 μL of water, and PNGase F (5 μL or 16 Takara mU) were added to the mixture and incubated at 37°C for 16 h. The digest was then loaded onto a solid phase extraction cartridge (Sep-Pak C18, 50 mg; Waters Corp., Milford, MA, USA) and washed with 1 mL of water. The flow-through and washings were combined and concentrated. For glycopeptidase A digestion, HRP (200 μg in water) was heated at 95°C for 10 min and then cooled to room temperature followed by digestion with pepsin (10 μg) in 5% formic acid at 37°C for 16 h. After the reaction was stopped by heating at 95°C for 10 min, the reaction mixture was dried *in vacuo*. The obtained residue was dissolved in 20 μL of 20 mM citrate buffer (pH 5.0) and glycopeptidase A (0.5 mU) was added to the solution. After incubation at 37°C for 16 h, the mixture was treated with a Sep-Pak C18 and concentrated as described above for PNGase F digestion.

### 2-AB labeling

The glycans and oximes were fluorescently labeled with 2-AB using 2-picoline borane, as previously described [22,23]. Briefly, 25 μL of 1 M 2-AB and 0.24 M 2-picoline borane in 10% acetic acid in methanol were added to the sample solution, and the dried sample was dissolved in 25 μL of 10% acetic acid. The mixture was incubated at 50°C for 3 h, loaded onto a Glycoclean S cartridge (Prozyme Inc., Hayward, CA, USA), and washed with 1 mL of 95% acetonitrile. The 2-AB-labeled glycans were recovered by eluting with 50 μL of water.

### HPLC

The 2-AB-labeled glycan mixture was analyzed using a Shimadzu LC-10A HPLC system equipped with an RF-20A XS fluorescence detector (Shimadzu Corp., Kyoto, Japan). Separations of 2-AB glycans by normal-phase HPLC were performed on a TSK gel Amide-80 column (5 μm, 4.6 × 250 mm; Tosoh Corp., Tokyo, Japan) with fluorescent detection at wavelengths of: Ex 330 nm and Em 420 nm. The samples were injected onto the column equilibrated with 36% solvent B (0.05% TFA in water) in solvent A (0.05% TFA in acetonitrile). The 2-AB glycans were separated with a linear gradient of 36–48% solvent B at a flow rate of 0.8 mL/min for 40 min at 45°C.

### Mass spectrometry (MS)

MS spectra were acquired using a matrix-assisted laser desorption ionization time-of-flight (MALDI-TOF) mass spectrometer (Ultraflex; Bruker Daltonik, Bremen, Germany). Ions were generated using a 337-nm nitrogen laser and were accelerated to 20 kV for reflectron mode and to 25 kV for linear mode. All spectra were obtained using a positive ion mode with delayed extraction. For MS/MS spectra acquisition, a MALDI-quadrupole ion trap (QIT)-TOF mass spectrometer (AXIMA-QIT; Shimadzu Corp., Kyoto, Japan) was used. Argon gas was used as a collision gas for collision-induced dissociation. For sample preparation, 0.5 μL of 2,5-dihydroxybenzoic acid (2,5-DHB; 10 mg/mL) in 30% ethanol was spotted onto a target plate (MTP 384 target plate ground steel [Bruker Daltonik] for Ultraflex and 384 specular surface stainless steel plate [Shimadzu Corp.] for AXIMA-QIT) and dried. For sialoglycans, at 95:5 mixture of 2,5-DHB and 2,5-DHB-Na was used as a matrix. Subsequently, an aliquot (0.5 μL) of the glycan solution was spotted onto the matrix crystal and dried.

## Results and discussion

### Overview of chemical de-N-glycosylation and derivatization

**Fig 1:**
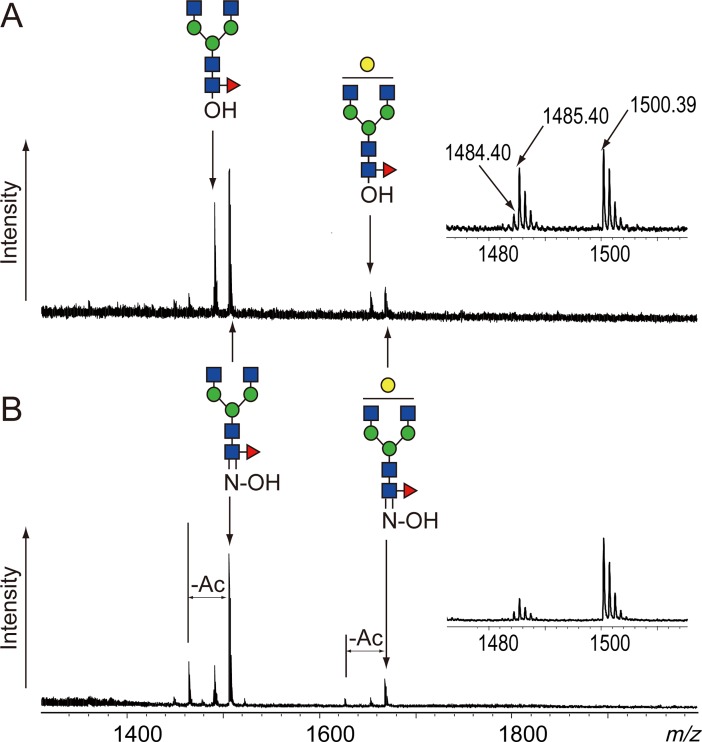
MS spectra of the alkaline de-N-glycosylation products of MAB-1-L001. (A) Products obtained from the reaction in 5% hydroxylamine; (B) products obtained from the reaction in 20% hydroxylamine. Both reactions were executed in hydroxylamine and 200 mM LiOH (final concentration) at 80°C for 1 h. The inserts show enlarged spectra of G0F(oxime and free glycan). G0F: fucosyl agalacto bi-antennary N-linked glycan; G1F: fucosyl monogalacto bi-antennary N-linked glycan. Glycan structures are depicted by using the CFG graphical notation for glycans (http://www.functionalglycomics.org/static/consortium/CFGnomenclature.pdf).

As shown in previous reports, N-linked glycans can be released from glycoproteins by heating to 80–95°C under alkaline conditions [19,20]. Chemical de-N-glycosylation is considered alkaline hydrolysis of the glycosyl carboxamide of Asn. The released glycans, however, easily decompose under alkaline conditions; therefore, fewer glycans than expected are obtained. Glycosylamines, which can be prepared in situ during deglycosylation in the presence of highly concentrated ammonia, are more stable in alkaline conditions than the corresponding reducing glycans [21,24]. By adding 25% aqueous ammonia solution to the alkaline lithium hydroxide de-N-glycosylation solution, we obtained the released glycans from a monoclonal antibody, MAB-1-L001, used as a model glycoprotein. The amount of obtained glycans, however, was still significantly lower than that obtained by PNGase F digestion (S1 Fig). Two possible reasons are suggested for such a low yield, i.e., insufficient deglycosylation and degradation of the released glycans. Hydroxylamine reacts with reducing glycans under acidic or alkaline conditions to afford the corresponding oximes, which are much more stable than glycosylamines under alkaline conditions. First, we examined the stabilization effect of hydroxylamine on the alkaline de-N-glycosylation of MAB-1-L001 in 0.1 M LiOH at 80°C for 1 h. MS spectra of the obtained glycans showed that the reaction afforded free glycans and the corresponding oximes (Fig 1). The oxime signals became greater, whereas the free glycan signals decreased inversely with increasing hydroxylamine concentration from 5% to 20% (Fig 1A and 1B). Deacetylation was also observed in the spectra. Hydroxylamine may partially decompose the acetamide group of GlcNAc by transamidation during the reaction. The signal of a free glycan (*m/z* 1485) was accompanied by a small signal, *m/z* 1484, that was attributed to glycosylamine. Thus, the chemical de-N-glycosylation using hydroxylamine released N-linked glycans from glycoproteins and afforded free glycans, glycosylamines, and oximes. Because the latter two species are relatively stable under alkaline conditions, degradation of glycans can be avoided.

**Fig 2:**
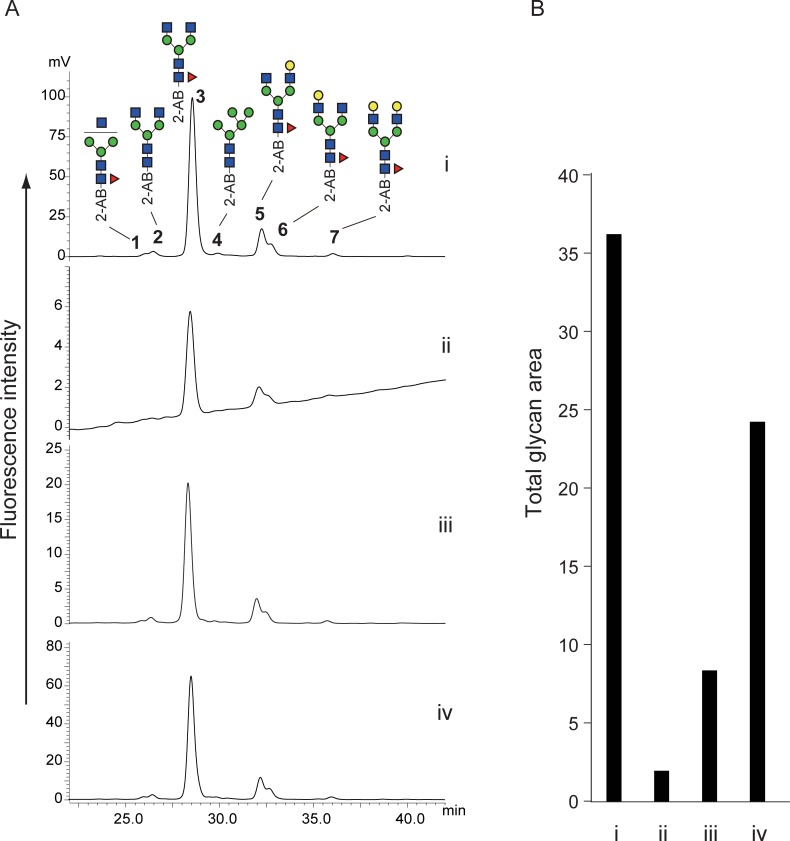
Comparison between alkaline de-N-glycosylation at 80°C for 1 h and PNGase F digestion. (A) HPLC chromatograms of PNGase F digestion (i), alkaline de-N-glycosylation without hydroxylamine (ii), alkaline de-N-glycosylation with 5% hydroxylamine (iii), and alkaline de-N-glycosylation with 20% hydroxylamine (iv). (B) Comparison of glycan recovery. Bar graph indicates sum of all glycan peak areas in the HPLC chromatograms. All chromatograms were obtained by injection of equal volumes of glycan solution prepared from the same quantity of glycoproteins. The numbered peaks were determined by MS and MS/MS. Glycan structures are depicted using CFG graphical notation for glycans (http://www.functionalglycomics.org/static/consortium/CFGnomenclature.pdf).

After clean up using a graphite carbon cartridge, the obtained oxime mixture containing glycosylamine and free glycans was subjected to reductive amination with 2-AB using 2-picoline borane [22,23]. 2-AB-labeled glycans were analyzed by HPLC with fluorescent detection (Fig 2). Chemical de-N-glycosylation afforded similar chromatograms to that obtained by PNGase F digestion (Fig 2A-i). Glycosylamines were easily hydrolyzed under the mild acidic conditions of the reductive amination into free glycans that should be 2-AB labeled in the process. These results suggest that oximes were also converted to 2-AB glycans. Anilines accelerate transimination under acidic conditions [24]. Therefore, 2-AB, an aniline derivative, may accelerate the conversion of an oxime into its corresponding imine derivative. All peaks obtained from the chemical de-N-glycosylation were fractionated and confirmed by MALDI-TOF MS and MALDI-QIT-TOF MS (Table 1 and S2–S8 Figs). The recovery rates of the reactions without hydroxylamine or with 5% hydroxylamine and 20% hydroxylamine compared with PNGase F digestion were 5%, 23% and 67%, respectively (Fig 2B). Higher concentrations of hydroxylamine accelerated oxime formation, as shown in Fig 1, and may increase recovery rates through stabilization of released glycans.

**Table 1:** Assignment of the peaks in the HPLC chromatogram of N-glycans of MAB-1-L001 by MALDI-TOF MS.

Glycoprotein	Peak no.	Obsd *(m/z*)	Calcd *(m/z*)^a^	Monosaccharide composition
MAB-1-L001	1	1402.51	1402.52	(HexNAc)_1_ (Deoxyhexose)_1_ + (Man)_3_(GlcNAc)_2_
	2	1459.52	1459.55	(HexNAc)_2_ + (Man)_3_(GlcNAc)_2_
	3	1605.66	1605.60	(HexNAc)_2_ (Deoxyhexose)_1_ + (Man)_3_(GlcNAc)_2_
	4	1377.83	1377.49	(Hex)_2_ + (Man)_3_(GlcNAc)_2_
	5	1767.67	1767.66	(Hex)_1_ (HexNAc)_2_ (Deoxyhexose)_1_ + (Man)_3_(GlcNAc)_2_
	6	1767.68	1767.66	(Hex)_1_ (HexNAc)_2_ (Deoxyhexose)_1_ + (Man)_3_(GlcNAc)_2_
	7	1929.55	1929.71	(Hex)_2_ (HexNAc)_2_ (Deoxyhexose)_1_ + (Man)_3_(GlcNAc)_2_

^a^ Values were calculated as sodium-adducted ion of 2-AB-labeled glycans.

**Fig 3:**
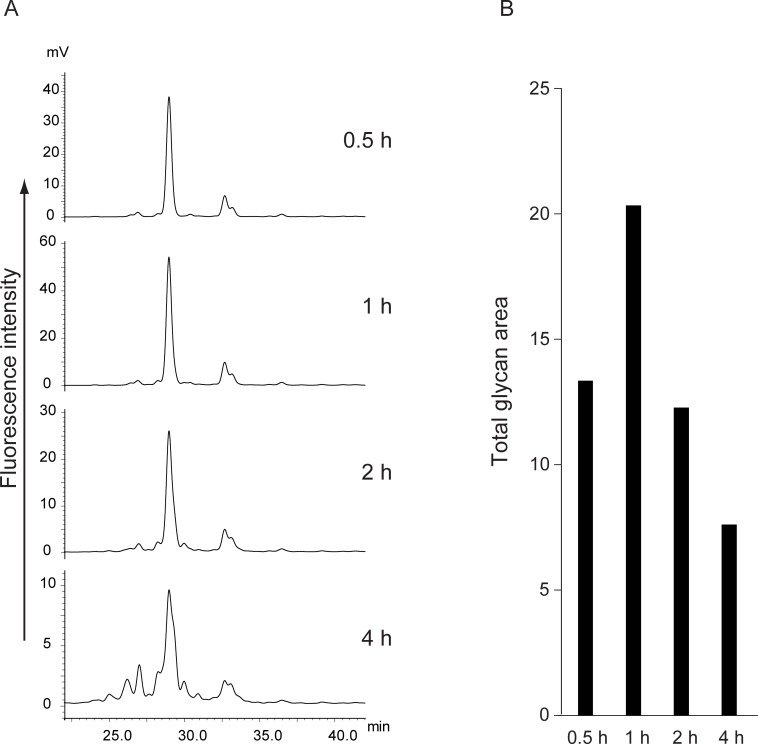
Time course of alkaline de-N-glycosylation with 20% hydroxylamine at 80°C. (A) HPLC chromatograms of 2-AB-labeled N-linked glycans released by 0.5 h-reaction, 1 h-reaction, 2 h-reaction, and 4 h-reaction. (B) Comparison of glycan recovery. Bar graph indicates sum of all glycan peak areas in the HPLC chromatograms. All chromatograms were obtained by injection of equal volumes of glycan solution prepared from the same quantity of glycoproteins.

**Fig 4:**
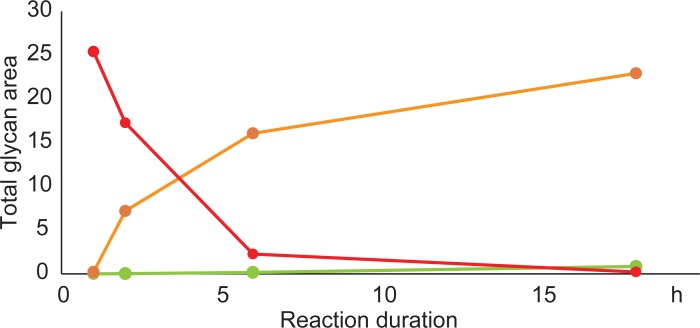
Comparison of time courses of alkaline de-N-glycosylation at different temperatures. Sum of all glycan peak areas in the HPLC chromatograms are plotted in red (80°C), orange (50°C), and green (25°C). The chromatograms are presented in S9 Fig. All chromatograms were obtained by injection of equal volumes of glycan solution prepared from the same quantity of glycoproteins.

We then examined the time course of the reaction using 20% hydroxylamine at 80°C. As shown in Fig 3, the recovery increased until 1 h but then gradually decreased over 1 h. After 4 h, the fluorescence intensities of the released glycans were reduced to approximately one-third of that obtained with a 1-h reaction, and small peaks that were absent in the PNGase F reaction appeared. These peaks were assumed to be degradation products, including deacetylation and the retro-aldol reaction [25]. To suppress the degradation, we examined the reactions at decreased temperatures. The degradation could be retarded by reducing the temperature, resulting in significant suppression of deglycosylation (Fig 4). Moreover, the degradation products were observed after 6 h and 18 h of the reaction, even at 50°C (S9 Fig). Thus, our chemical de-N-glycosylation afforded the best result at 80°C for 1 h in the presence of 20% hydroxylamine. The reasons for the lower recovery rate compared with the PNGase F digestion are currently unclear; however, over-reaction causing degradation may be one main cause.

### Compatibility with NeuGc

**Fig 5:**
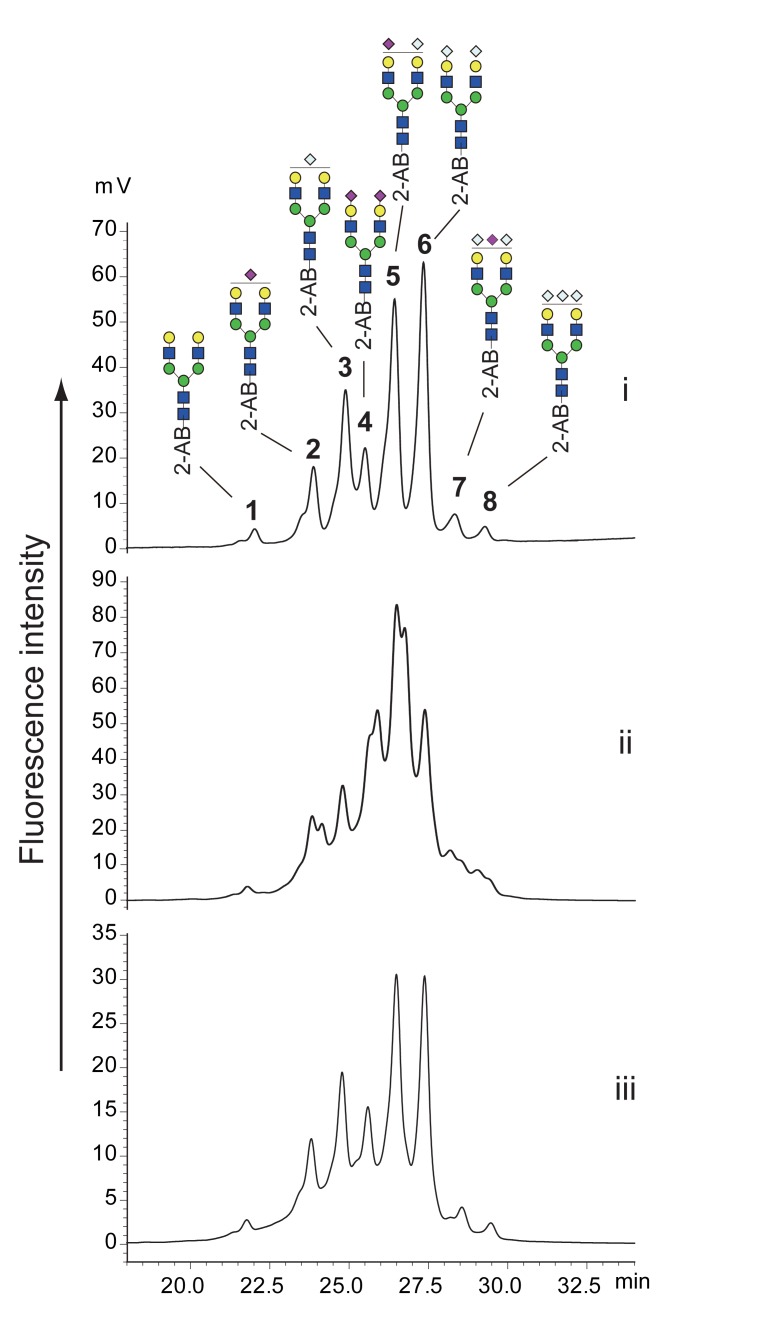
Comparison of HPLC profiles of 2-AB-labeled N-linked glycans released from bovine apo-transferrin by various conditions. HPLC chromatograms of PNGase F digestion (i), alkaline de-N-glycosylation with 20% hydroxylamine at 80°C for 1 h (ii), and alkaline de-N-glycosylation with 20% hydroxylamine at 50°C for 1 h (iii). All chromatograms were obtained by injection of equal volumes of glycan solution prepared from the same quantity of glycoproteins. The numbered peaks were determined by MS. Glycan structures are depicted by using the CFG graphical notation for glycans (http://www.functionalglycomics.org/static/consortium/CFGnomenclature.pdf).

**Fig 6:**
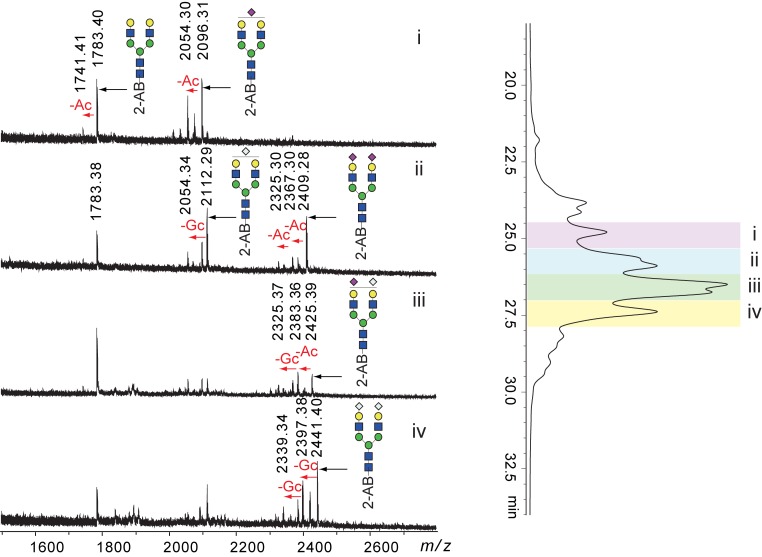
Deacylation of N-linked glycans in apo-transferrin during alkaline de-N-glycosylation at 80°C for 1 h. MS spectra of indicated fractions (i–iv) in the HPLC chromatogram of Fig 5-ii. Glycan structures are depicted by using the CFG graphical notation for glycans (http://www.functionalglycomics.org/static/consortium/CFGnomenclature.pdf).

Hydrazinolysis decomposes all glycan amide groups, including GlcNAc, GalNAc, NeuAc, and NeuGc. The obtained glycans were re-acetylated after hydrazinolysis. Therefore, glycans containing NeuGc should be detected as the corresponding glycans containing NeuAc. We examined whether our alkaline de-N-glycosylation method could liberate glycans containing NeuGc without amide decomposition. As a model glycoprotein, we used bovine apo-transferrin, which had bi-antennary N-linked glycans containing both NeuAc and NeuGc. Apo-transferrin was deglycosylated by PNGase F and alkaline de-N-glycosylation at 80°C for 60 min. The obtained glycans were labeled with 2-AB and separated by chromatography using a normal phase (Amide-80) column. HPLC chromatograms of PNGase F digestion and alkaline de-N-glycosylation are shown in Fig 5i and 5ii, respectively. Each peak in Fig 5-i was fractionated and analyzed by MALDI MS (Table 2 and S10–S17 Figs). Alkaline de-N-glycosylation at 80°C for 60 min afforded a complicated mixture of bi-antennary sialoglycans containing NeuAc and NeuGc, along with NeuNH_2,_ which formed because of the removal of acyl groups from NeuAc and NeuGc (Fig 5-ii). The deacylation was confirmed by MS of the fractions obtained by HPLC (Fig 6). These results showed that amides of sialic acids were partially degraded during the chemical de-N-glycosylation. Amides at the 5-position of sialic acid may be more susceptible to these conditions than amides at the 2-position of HexNAc because neutral glycans were observed intact following HPLC, as shown in Fig 2. In addition, MS spectra of the degradation products also indicated differences in the susceptibilities of acyl groups between sialic acid and HexNAc (Fig 6). Glycosidic linkages of sialic acids are easily decomposed by in-sourced decay (ISD) during the MALDI process [26]. An asialo bi-antennary glycan (*m/z* 1783) was observed in MS spectra of all degraded fractions due to ISD. A monosialoglycan with NeuAc (*m/z* 2096) was accompanied by a deacetylated form (*m/z* 2054) with comparable intensity, whereas the asialo glycan (*m/z* 1783) was accompanied by a small signal of the corresponding deacetylated glycan (*m/z* 1741), even though the glycan contained four amide groups of GlcNAcs. Glycans containing NeuGc also provided similar results (Fig 6-iv). Deacylation during alkaline de-N-glycosylation was avoided by reducing the reaction temperature. Although each peak was broadened, the glycans obtained by the reaction at 50°C for 60 min gave almost the same glycan profile as that obtained by PNGase F (Fig 5-iii). The peak broadening may have been due to slight deacylation of sialic acids. The glycan recovery, however, was approximately 50% of that obtained by the PNGase F reaction when the alkaline de-N-glycosylation was performed at 50°C.

**Table 2:** Assignment of the peaks in HPLC chromatogram of N-glycans of apo-transferrin by MALDI-TOF MS.

Glycoprotein	Peak no.	Obsd *(m/z*)	Calcd *(m/z*)^a^	Monosaccharide composition
Apo-transferrin (bovine)	1	1783.62	1783.65	(Gal)_2_ (GlcNAc)_2_ + (Man)_3_(GlcNAc)_2_
	2	2096.56	2096.73	(Gal)_2_ (GlcNAc)_2_ (NeuAc+Na)_1_ + (Man)_3_(GlcNAc)_2_
	3	2112.72	2112.72	(Gal)_2_ (GlcNAc)_2_ (NeuGc+Na)_1_ + (Man)_3_(GlcNAc)_2_
	4	2409.61	2409.81	(Gal)_2_ (GlcNAc)_2_ (NeuAc+Na)_2_ + (Man)_3_(GlcNAc)_2_
	5	2425.79	2425.80	(Gal)_2_ (GlcNAc)_2_ (NeuAc+Na)_1_ (NeuGc+Na)_1_ + (Man)_3_(GlcNAc)_2_
	6	2441.77	2441.80	(Gal)_2_ (GlcNAc)_2_ (NeuGc+Na)_2_ + (Man)_3_(GlcNAc)_2_
	7	2688.56	2688.92	(Gal)_2_ (GlcNAc)_2_ (NeuGc)_2_(NeuAc)_1_ + (Man)_3_(GlcNAc)_2_
	8	2705.20	2704.92	(Gal)_2_ (GlcNAc)_2_ (NeuGc)_3_ + (Man)_3_(GlcNAc)_2_

^a^ Values were calculated as sodium-adducted ion of 2-AB-labeled glycans.

### Compatibility with N-linked glycans from plants

**Fig 7:**
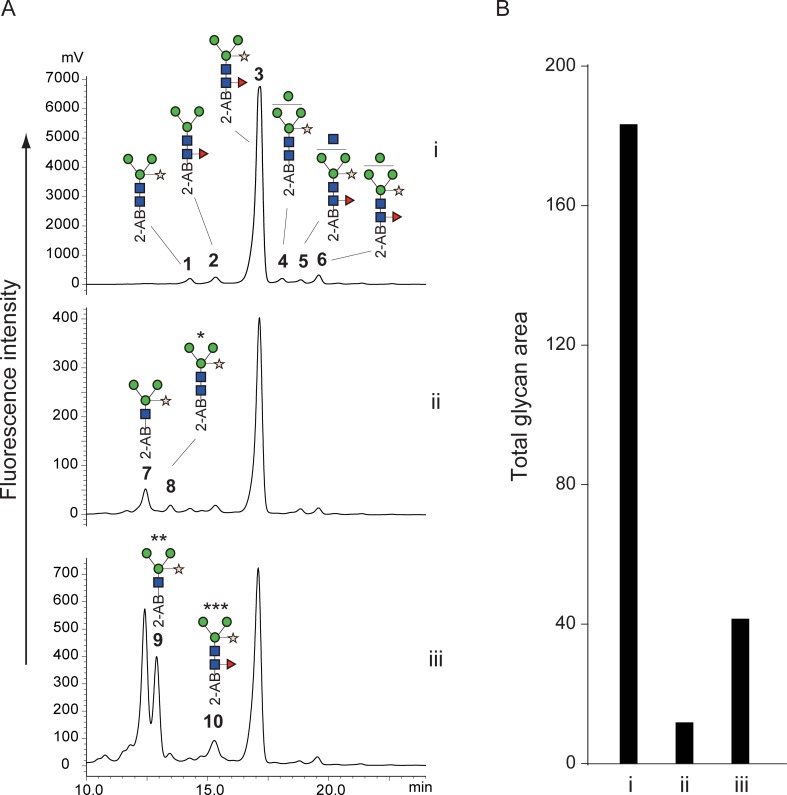
Comparison of HPLC profiles of 2-AB-labeled N-linked glycans released from HRP by various conditions. (A) HPLC chromatograms of glycopeptidase A digestion (i), alkaline de-N-glycosylation with 20% hydroxylamine at 50°C for 1 h (ii), and alkaline de-N-glycosylation with 20% hydroxylamine at 80°C for 1 h (iii). (B) Comparison of glycan recovery. Bar graph indicates sum of all glycan peak areas in the HPLC chromatograms. All chromatograms were obtained by injection of equal volumes of glycan solution prepared from the same quantity of glycoproteins. The numbered peaks were determined by MS and MS/MS and are summarized in Table 3. Glycan structures are depicted by using CFG graphical notation for glycans (http://www.functionalglycomics.org/static/consortium/CFGnomenclature.pdf).

**Fig 8:**
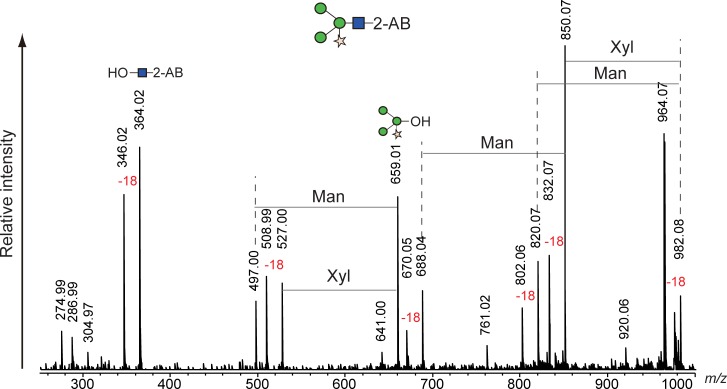
MS/MS spectrum of degraded peak 7 appearing during alkaline de-N-glycosylation of HRP at 80°C for 1 h.

**Fig 9:**
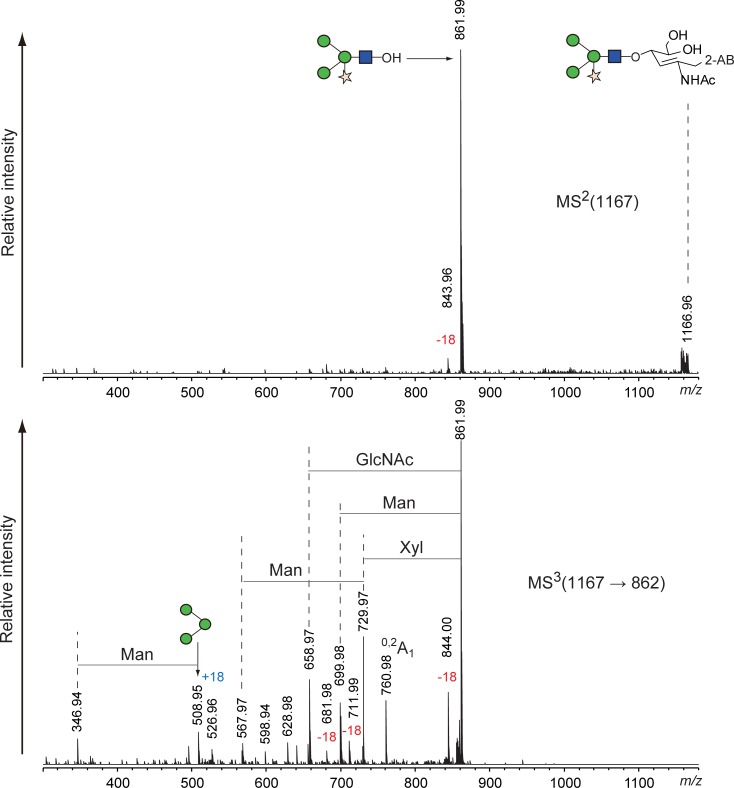
MS^n^ spectrum of degraded peak 8 appearing during alkaline de-N-glycosylation of HRP at 80°C for 1 h.

A Fuc α1–3 GlcNAc structure at the reducing end of N-linked glycans is usually found in glycoproteins of plant and insect origin [27,28]. These glycans are resistant to digestion by PNGase F but not by glycopeptidase A, which is also expensive and requires protein denaturation and/or protease digestion prior to the enzyme digestion. Hydrazinolysis is applicable to glycoproteins regardless of the presence of Fuc α1–3 GlcNAc structures. However, fucose at the 3-position of the reducing end of GlcNAc is easily eliminated during hydrazinolysis in insufficient anhydrous conditions [29]. We examined whether our alkaline de-N-glycosylation method could liberate glycans containing Fuc α1–3 GlcNAc at the reducing end without elimination of the fucose. As a model glycoprotein, we used HRP whose N-linked glycans contained the α1–3 linkage of fucose at the reducing end. HRP was subjected to conventional glycopeptidase A digestion and our chemical de-N-glycosylation at 50°C and 80°C for 1 h, and the obtained glycans were then labeled with 2-AB and chromatographed with normal-phase HPLC. The HPLC chromatograms are shown in Fig 7, and each peak, including the small ones, was fractionated and assigned by MALDI MS and MS/MS (Table 3 and S18–S27 Figs). Glycopeptidase A digestion of HRP correlated with published data [27,28]. The recovery rates of the chemical de-N-glycosylation were significantly lower than those of glycopeptidase A digestion, compared to the experiments using the MAB-L001 antibody and apo-transferrin. The HPLC chromatogram of chemical de-N-glycosylation at 50°C, however, was similar to that of glycopeptidase A digestion (Fig 7A-ii). In a careful review of the chromatograms, we found small peaks 7 and 8 that were observed only in chemical de-N-glycosylation and not in glycopeptidase A digestion. MS/MS analysis revealed that the small peaks represented a glycan with only one GlcNAc and a Fuc-eliminated glycan (Figs 8 and 9). Such glycans have been reported as degradation products during chemical deglycosylation of plant glycoproteins containing Fuc α1–3 GlcNAc structures [29]. Glycan 7 was largely increased by the chemical de-N-glycosylation at 80°C, accompanied by an unknown isomer 9 with comparable intensity. The unreduced product 10 of the major glycan 3 was also detected (Fig 7A-iii). In contrast, glycan 4 was only observed in the HPLC chromatogram of glycopeptidase A digestion. The reason for this finding is unclear, but some sites among the eight N-glycosylated sites of HRP may be resistant to chemical de-N-glycosylation. Thus, our alkaline de-N-glycosylation for a plant glycoprotein afforded a better result following reaction at 50°C, despite only 7% recovery compared with glycopeptidase A digestion. However, the degradation of glycans containing Fuc α1–3 GlcNAc at the reducing end could not be avoided.

**Table 3:** Assignment of the peaks in HPLC chromatogram of N-glycans of HRP by MALDI-TOF MS.

Glycoprotein	Peak no.	Obsd *(m/z*)	Calcd *(m/z*)^a^	Monosaccharide composition
Horseradish peroxidase	1	1185.49	1185.43	(Man)_3_ (GlcNAc)_2_ (Xyl)_1_
	2	1199.51	1199.44	(Man)_3_ (GlcNAc)_2_ (Fuc)_1_
	3	1331.42	1331.49	(Man)_3_ (GlcNAc)_2_ (Fuc)_1_ (Xyl)_1_
	4	1347.55	1347.48	(Man)_4_ (GlcNAc)_2_ (Xyl)_1_
	5	1534.25	1534.57	(Man)_3_ (GlcNAc)_3_ (Fuc)_1_ (Xyl)_1_
	6	1493.28	1493.54	(Man)_4_ (GlcNAc)_2_ (Fuc)_1_ (Xyl)_1_
	7	982.38	982.35	(Man)_3_ (GlcNAc)_1_ (Xyl)_1_
	8	1167.43	1167.42	(Man)_3_ (GlcNAc)_2_ (Xyl)_1_ -H_2_O
	9	982.40	982.35	(Man)_3_ (GlcNAc)_1_ (Xyl)_1_
	10^b^	1329.53	1329.47	(Man)_3_ (GlcNAc)_2_ (Fuc)_1_ (Xyl)_1_

^a^ Values were calculated as sodium-adducted ion of 2-AB-labeled glycans.

^b^ Unreduced 2-AB labeled glycan.

## Conclusion

Here, we have presented a chemical de-N-glycosylation method that uses hydroxylamine under alkaline conditions at 50˚C and 80˚C. Hydroxylamine functions as a stabilizer for the released glycans under aqueous alkaline conditions by forming oximes. The released glycans were obtained as a mixture of oximes, glycosylamines, and free glycans that could all be labeled with 2-AB by reductive amination. Other fluorescent amines, including 2-AA, PA, AMAC, and APTS, which are often used for glycan analysis with HPLC and CE, may also be applicable to the tagging of the released glycan mixture. In contrast to hydrazinolysis, our method does not require anhydrous conditions and extreme care in handling or re-acetylation. Notably, over-reactions including deamidation of HexNAc and sialic acid, and “peeling” of Fuc α1–3 GlcNAc at the reducing end [29], were observed in the case of longer reaction duration and increased reaction temperature. Deamidation of HexNAc and sialic acid was almost avoided by termination within 1 h of the reaction at 80°C and 50°C, respectively. However, “peeling” of Fuc α1–3 GlcNAc was observed in the reaction at 50°C for 1 h. Therefore, the chemical de-N-glycosylation should be carefully applied to glycoproteins from insect and plant sources. In this study, we used LiOH as an alkaline catalyst. The possibility of suppression of the over-reaction by replacing the catalyst with another mild alkaline will be pursued in the future. The recovery rates of glycans liberated by the presented chemical de-N-glycosylation depend on reaction temperature, reaction duration, and substrates. For the therapeutic antibody, the recovery reached a maximum of 67% of conventional enzymatic deglycosylation, whereas the recovery including degradation products was 22% for HRP in the same reaction condition. The reasons for the differences are currently unknown. Increasing the temperature and reaction duration led to degradation, as described above, whereas decreasing these parameters resulted in lower release. Considering this balance, we proposed the use of 80°C for 1 h for asialo glycoproteins from mammals and 50°C for 1 h for sialoglycoproteins. We conclude that the rapid and simple chemical de-N-glycosylation method presented here will accelerate evaluation of the glycosylation of therapeutic antibodies produced using mammalian sources.

## Supporting information

S1 Fig Comparison of HPLC chromatogram between alkaline de-N-glycosylation with 25% ammonia and PNGaseF digestion. (A) PNGase F digestion, (B) alkaline de-N-glycosylation with 25% ammonia.
(EPS)

S2 Fig MS and MS/MS spectra of the glycan peak 1 of MAB-1-L001. (A) MS spectrum, (B) MS/MS spectrum.
(PDF)

S3 Fig MS and MS/MS spectra of the glycan peak 2 of MAB-1-L001. (A) MS spectrum, (B) MS/MS spectrum.
(PDF)

S4 Fig MS and MS/MS spectra of the glycan peak 3 of MAB-1-L001. (A) MS spectrum, (B) MS/MS spectrum.
(PDF)

S5 Fig MS and MS/MS spectra of the glycan peak 4 of MAB-1-L001. (A) MS spectrum, (B) MS/MS spectrum.
(PDF)

S6 Fig MS and MS/MS spectra of the glycan peak 5 of MAB-1-L001. (A) MS spectrum, (B) MS/MS spectrum.
(PDF)

S7 Fig MS and MS/MS spectra of the glycan peak 6 of MAB-1-L001. (A) MS spectrum, (B) MS/MS spectrum.
(PDF)

S8 Fig MS and MS/MS spectra of the glycan peak 7 of MAB-1-L001. (A) MS spectrum, (B) MS/MS spectrum.
(PDF)

S9 Fig Time courses of HPLC chromatograms of the alkaline de-N-glycosylation of MAB-1-L001 at different temperatures. (A) 25°C, (B) 50°C, (C) 80°C.
(EPS)

S10 Fig MS spectrum of the glycan peak 1 of apo-transferrin. (PDF)

S11 Fig MS spectrum of the glycan peak 2 of apo-transferrin. (PDF)

S12 Fig MS spectrum of the glycan peak 3 of apo-transferrin. (PDF)

S13 Fig MS spectrum of the glycan peak 4 of apo-transferrin. (PDF)

S14 Fig MS spectrum of the glycan peak 5 of apo-transferrin. (PDF)

S15 Fig MS spectrum of the glycan peak 6 of apo-transferrin. (PDF)

S16 Fig MS spectrum of the glycan peak 7 of apo-transferrin. (PDF)

S17 Fig MS spectrum of the glycan peak 8 of apo-transferrin. (PDF)

S18 Fig MS and MS/MS spectra of the glycan peak 1 of HRP. (A) MS spectrum, (B) MS/MS spectrum.
(PDF)

S19 Fig MS and MS/MS spectra of the glycan peak 2 of HRP. (A) MS spectrum, (B) MS/MS spectrum.
(PDF)

S20 Fig MS and MS/MS spectra of the glycan peak 3 of HRP. (A) MS spectrum, (B) MS/MS spectrum.
(PDF)

S21 Fig MS and MS/MS spectra of the glycan peak 4 of HRP. (A) MS spectrum, (B) MS/MS spectrum.
(PDF)

S22 Fig MS and MS/MS spectra of the glycan peak 5 of HRP. (A) MS spectrum, (B) MS/MS spectrum.
(PDF)

S23 Fig MS and MS/MS spectra of the glycan peak 6 of HRP. (A) MS spectrum, (B) MS/MS spectrum.
(PDF)

S24 Fig MS spectra of the glycan peak 7 of HRP. (PDF)

S25 Fig MS spectra of the glycan peak 8 of HRP. (PDF)

S26 Fig MS and MS/MS spectra of the glycan peak 9 of HRP. (A) MS spectrum, (B) MS/MS spectrum.
(PDF)

S27 Fig MS and MS/MS spectra of the glycan peak 10 of HRP. (A) MS spectrum, (B) MS/MS spectrum.
(PDF)
