# Supplementary material for: Rapid chemical de-N-glycosylation and derivatization for liquid chromatography of immunoglobulin N-linked glycans
Source: PLoS One. 2018 May 3;13(5):e0196800. doi: 10.1371/journal.pone.0196800 (PMC5933716; doi:10.1371/journal.pone.0196800)
Supplement: S2 Fig — (A) MS spectrum, (B) MS/MS spectrum. (PDF) [file pone.0196800.s002.pdf]

A

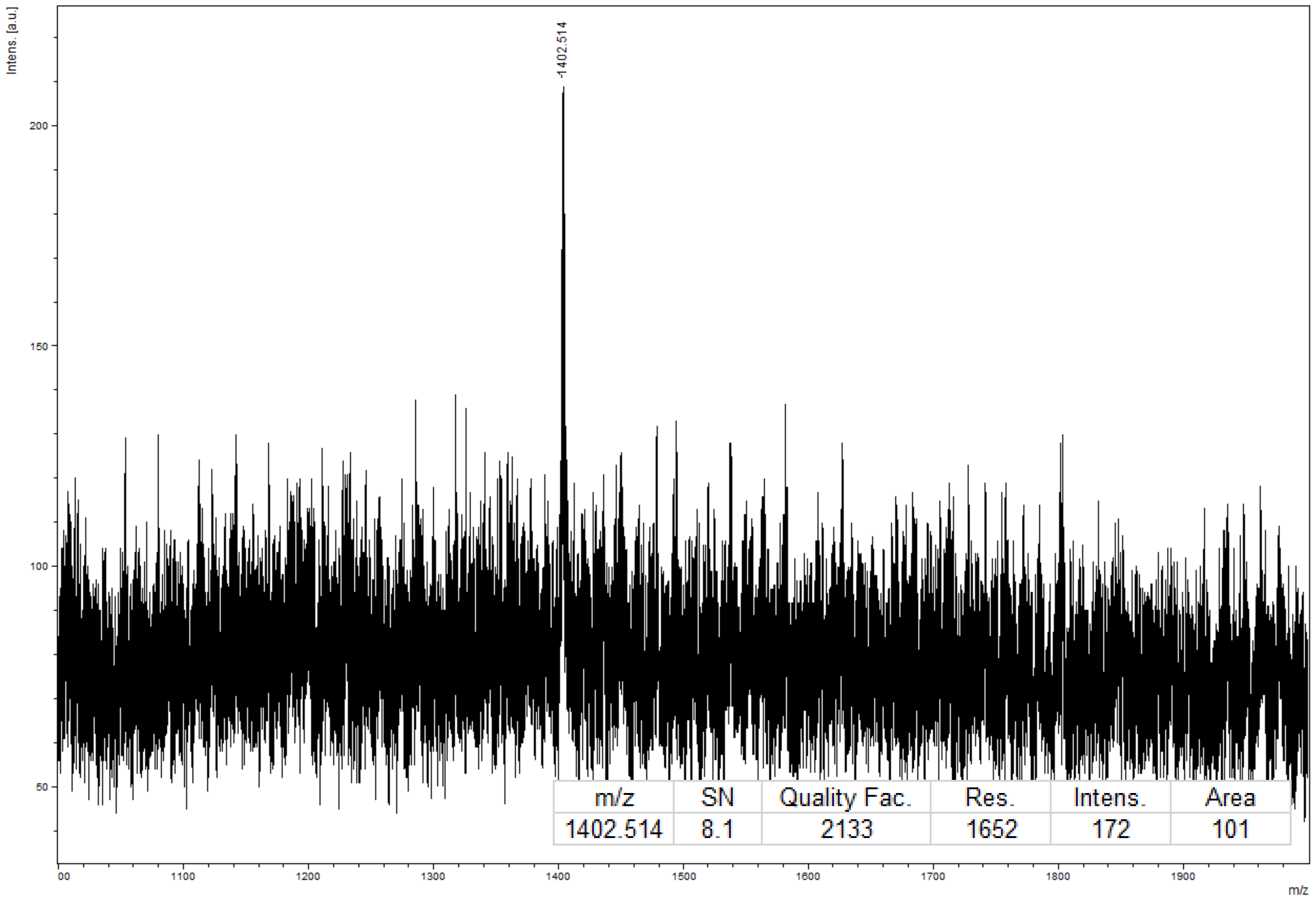

Intens. [a.u.]
1402.514
200
150
100
50
00
1100
1200
1300
1400
1500
1600
1700
1800
1900
m/z
m/z
SN
Quality Fac.
Res.
Intens.
Area
1402.514
8.1
2133
1652
172
101

B

2.8.5.20090629ver.R04_120615(S/N:U30014000002)

Data: 2018-02-21-LP110-CID165(1402)-peak10001.J15[c] 21 Feb 2018 11:59 Cal: 120817 6 Apr 2017 11:02 (CID of 1402.10)
Shimadzu Biotech Axima QIT 2.9.1.20100121: Mode positive, Mid 750+, Power: 110
%Int. 6.0 mV[sum= 3627 mV] Profiles 1-600 Unsmoothed

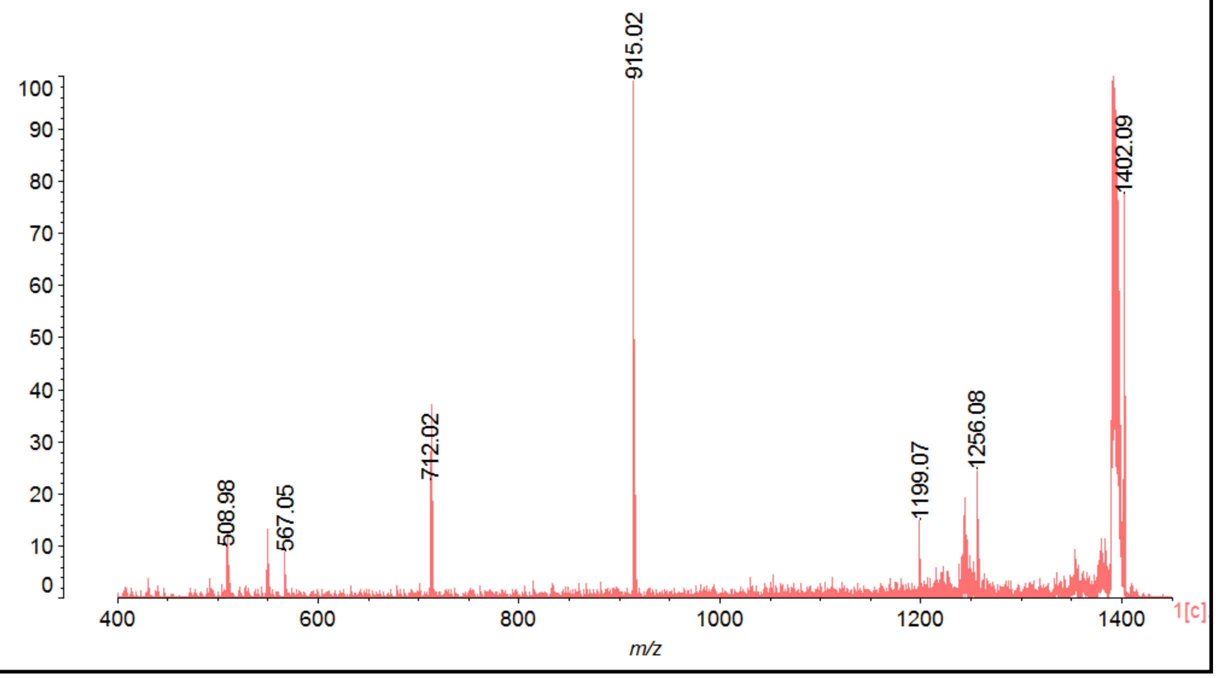


2.8.5.20090629ver.R04_120615(S/N:U30014000002)

Data: 2018-02-21-LP110-CID165(1402)-peak10001.J15[c] 21 Feb 2018 11:59 Cal: 12081
Shimadzu Biotech Axima QIT 2.9.1.20100121: Mode positive, Mid 750+, Power: 110

| Mass | %Area | %Total | Apex (mV) | Resolution | S / N | Flags |
|---|---|---|---|---|---|---|
| 508.98 | 8.44 | 3.32 | 0.58 | 0.00 | 0.00 | M |
| 549.03 | 3.99 | 1.57 | 0.32 | 0.00 | 0.00 | M |
| 567.05 | 4.23 | 1.67 | 0.52 | 0.00 | 0.00 | M |
| 712.02 | 21.06 | 8.28 | 1.35 | 0.00 | 0.00 | M |
| 915.02 | 87.41 | 34.38 | 5.99 | 0.00 | 0.00 | M |
| 1199.07 | 8.29 | 3.26 | 0.89 | 0.00 | 0.00 | M |
| 1256.08 | 20.84 | 8.20 | 1.47 | 0.00 | 0.00 | M |
| 1402.09 | 100.00 | 39.33 | 4.67 | 0.00 | 0.00 | M |
