# Supplementary material for: Rapid chemical de-N-glycosylation and derivatization for liquid chromatography of immunoglobulin N-linked glycans
Source: PLoS One. 2018 May 3;13(5):e0196800. doi: 10.1371/journal.pone.0196800 (PMC5933716; doi:10.1371/journal.pone.0196800)
Supplement: S3 Fig — (A) MS spectrum, (B) MS/MS spectrum. (PDF) [file pone.0196800.s003.pdf]

A

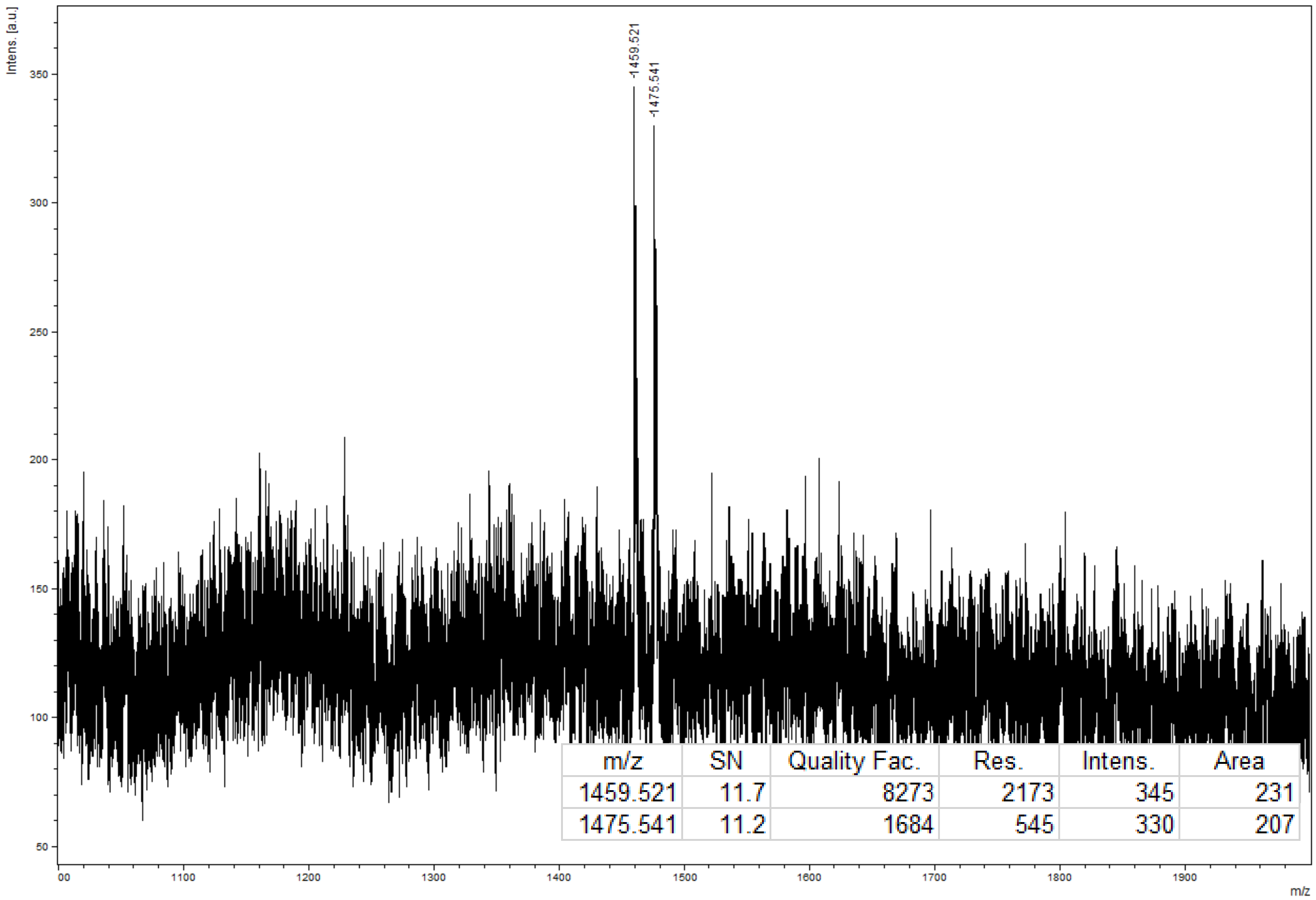

m/z	SN	Quality Fac.	Res.	Intens.	Area
1459.521	11.7	8273	2173	345	231
1475.541	11.2	1684	545	330	207

B

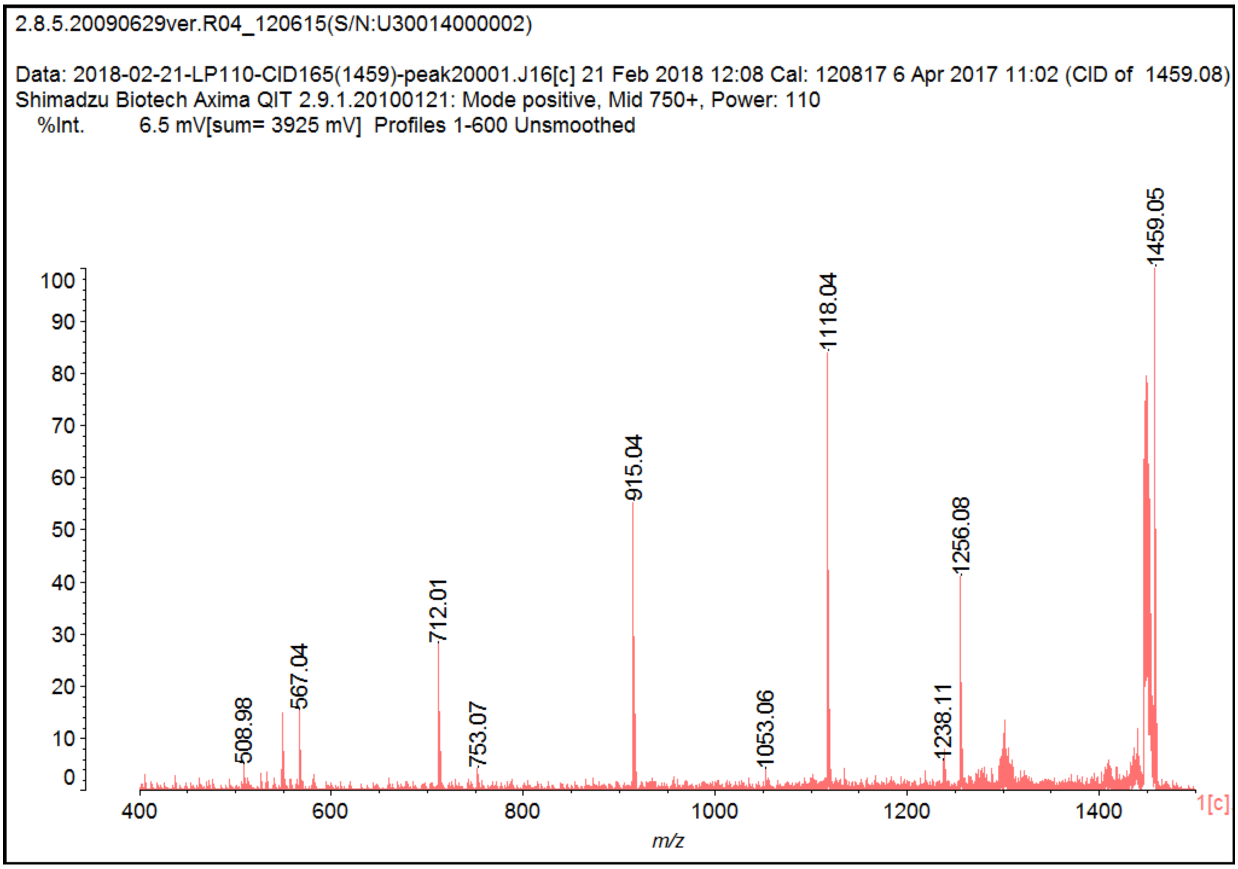

2.8.5.20090629ver.R04_120615(S/N:U30014000002)

Data: 2018-02-21-LP110-CID165(1459)-peak20001.J16[c] 21 Feb 2018 12:08 Cal: 12081
Shimadzu Biotech Axima QIT 2.9.1.20100121: Mode positive, Mid 750+, Power: 110

Mass	%Area	%Total	Apex (mV)	Resolution	S / N	Flags
508.98	4.10	1.43	0.31	0.00	0.00	M
550.00	14.21	4.94	0.97	0.00	0.00	M
567.04	12.77	4.44	0.99	0.00	0.00	M
712.01	27.87	9.69	1.82	0.00	0.00	M
753.07	4.13	1.44	0.26	0.00	0.00	M
915.04	53.64	18.64	3.61	0.00	0.00	M
1053.06	4.60	1.60	0.25	0.00	0.00	M
1118.04	100.00	34.75	5.48	0.00	0.00	M
1238.11	5.12	1.78	0.35	0.00	0.00	M
1256.08	52.57	18.27	2.68	0.00	0.00	M
1459.05	8.72	3.03	6.54	0.00	0.00	M
