# Supplementary material for: Rapid chemical de-N-glycosylation and derivatization for liquid chromatography of immunoglobulin N-linked glycans
Source: PLoS One. 2018 May 3;13(5):e0196800. doi: 10.1371/journal.pone.0196800 (PMC5933716; doi:10.1371/journal.pone.0196800)
Supplement: S4 Fig — (A) MS spectrum, (B) MS/MS spectrum. (PDF) [file pone.0196800.s004.pdf]

A

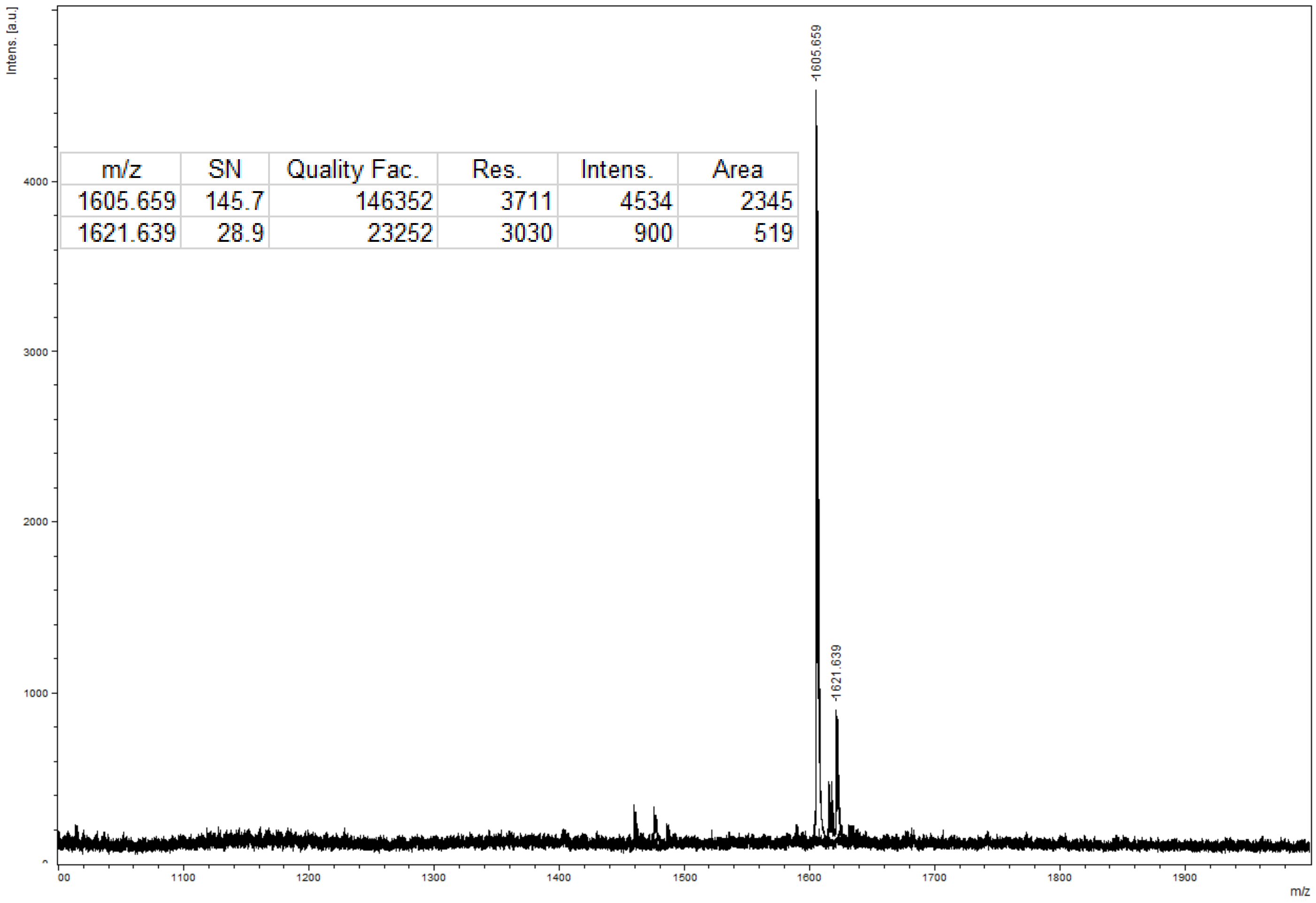

m/z	SN	Quality Fac.	Res.	Intens.	Area
1605.659	145.7	146352	3711	4534	2345
1621.639	28.9	23252	3030	900	519

B

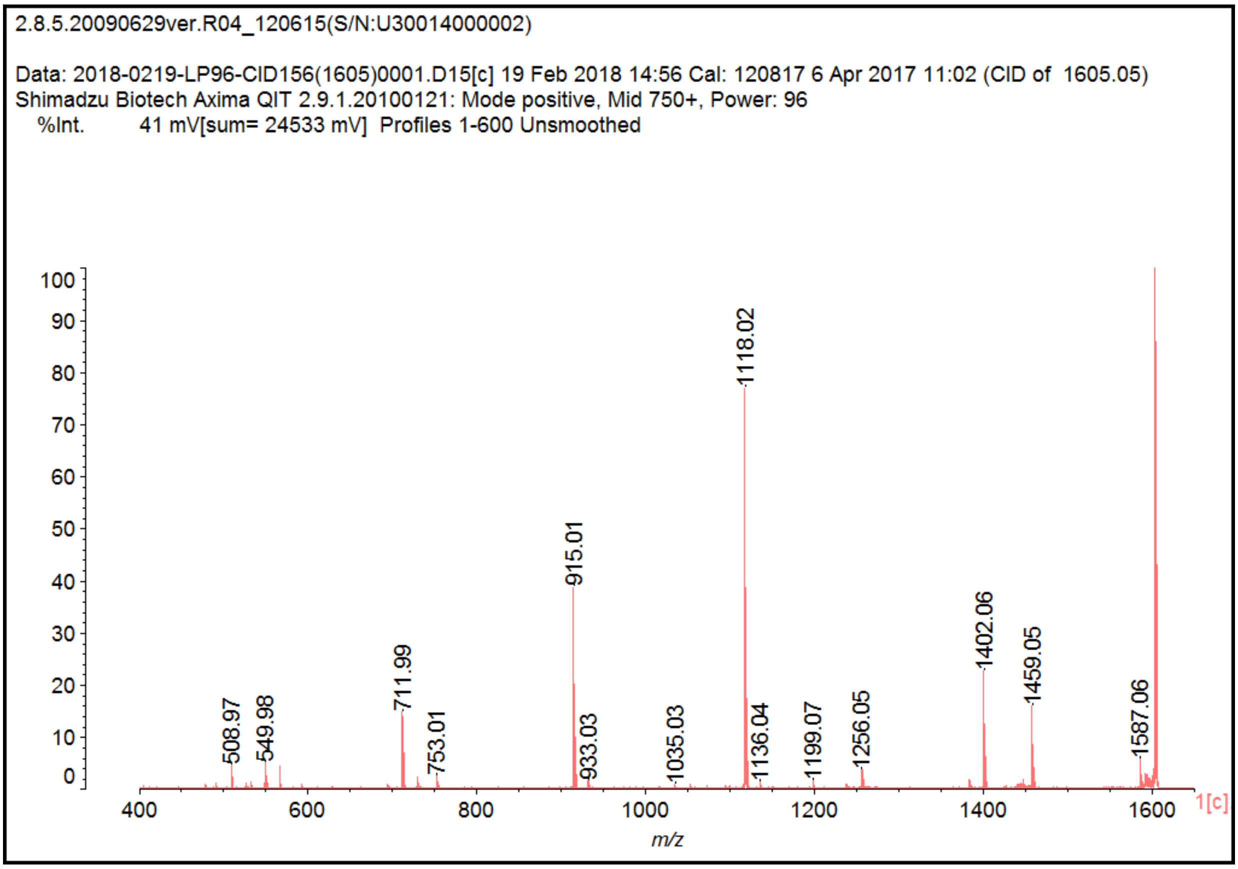

2.8.5.20090629ver.R04_120615(S/N:U30014000002)

Data: 2018-0219-LP96-CID156(1605)0001.D15[c] 19 Feb 2018 14:56 Cal: 120817 6 Apr 2
Shimadzu Biotech Axima QIT 2.9.1.20100121: Mode positive, Mid 750+, Power: 96

Mass	%Area	%Total	Apex (mV)	Resolution	S / N	Flags
508.97	4.68	1.84	1.88	0.00	0.00	M
531.98	1.24	0.49	0.50	0.00	0.00	M
549.98	5.06	1.99	2.01	0.00	0.00	M
567.03	4.44	1.75	1.79	0.00	0.00	M
711.99	17.50	6.90	6.05	0.00	0.00	M
729.99	1.74	0.68	0.89	0.00	0.00	M
753.01	2.74	1.08	1.01	0.00	0.00	M
915.01	45.53	17.94	15.82	0.00	0.00	M
933.03	1.82	0.72	0.59	0.00	0.00	M
1035.03	0.84	0.33	0.31	0.00	0.00	M
1118.02	100.00	39.40	31.43	0.00	0.00	M
1136.04	1.47	0.58	0.50	0.00	0.00	M
1199.07	2.24	0.88	0.66	0.00	0.00	M
1238.99	0.73	0.29	0.34	0.00	0.00	M
1256.05	3.76	1.48	1.45	0.00	0.00	M
1384.03	2.54	1.00	0.69	0.00	0.00	M
1402.06	29.44	11.60	9.21	0.00	0.00	M
1459.05	22.13	8.72	6.49	0.00	0.00	M
1587.06	5.92	2.33	2.35	0.00	0.00	M
