# Supplementary material for: Rapid chemical de-N-glycosylation and derivatization for liquid chromatography of immunoglobulin N-linked glycans
Source: PLoS One. 2018 May 3;13(5):e0196800. doi: 10.1371/journal.pone.0196800 (PMC5933716; doi:10.1371/journal.pone.0196800)
Supplement: S5 Fig — (A) MS spectrum, (B) MS/MS spectrum. (PDF) [file pone.0196800.s005.pdf]

A

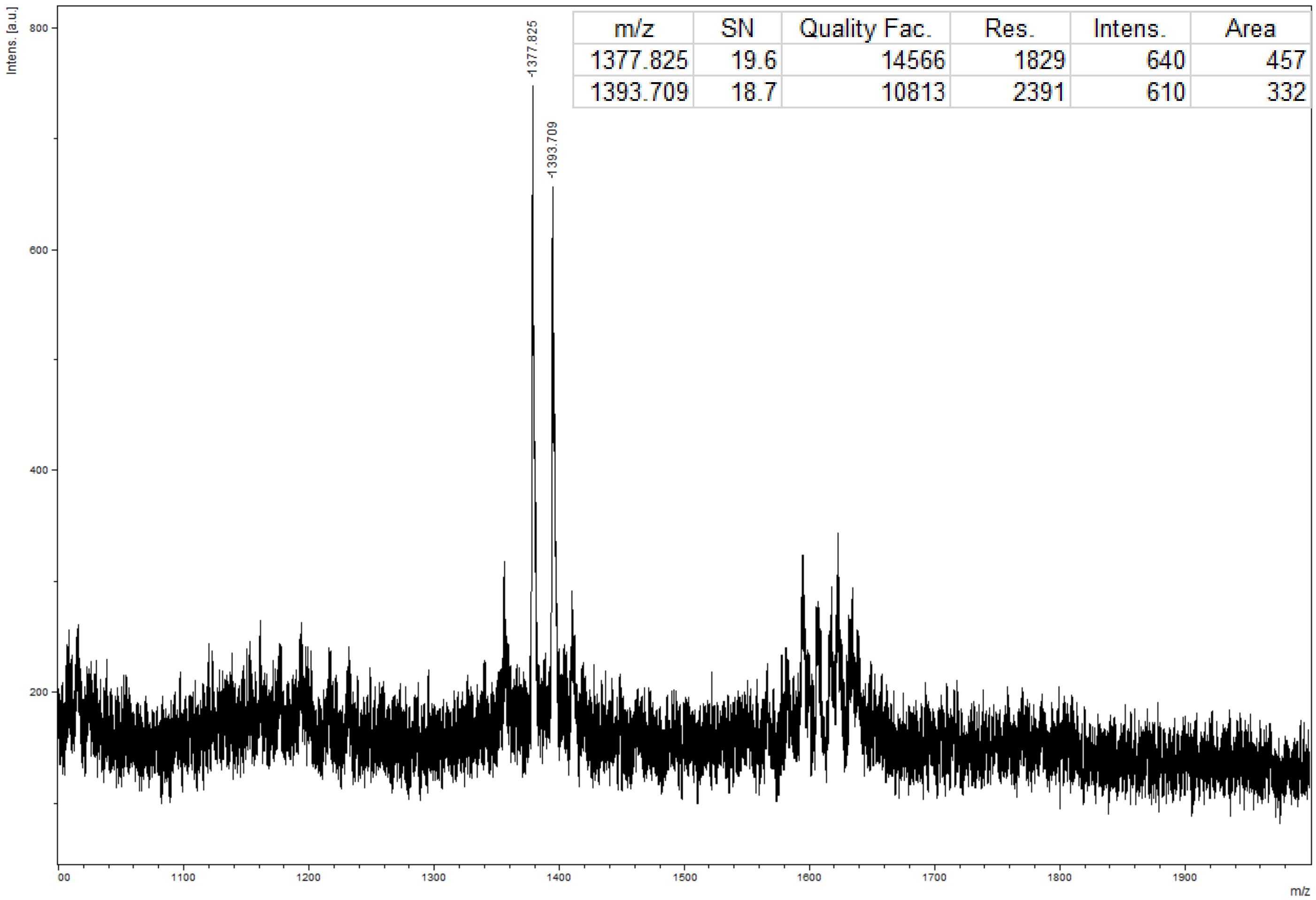

m/z	SN	Quality Fac.	Res.	Intens.	Area
1377.825	19.6	14566	1829	640	457
1393.709	18.7	10813	2391	610	332

B

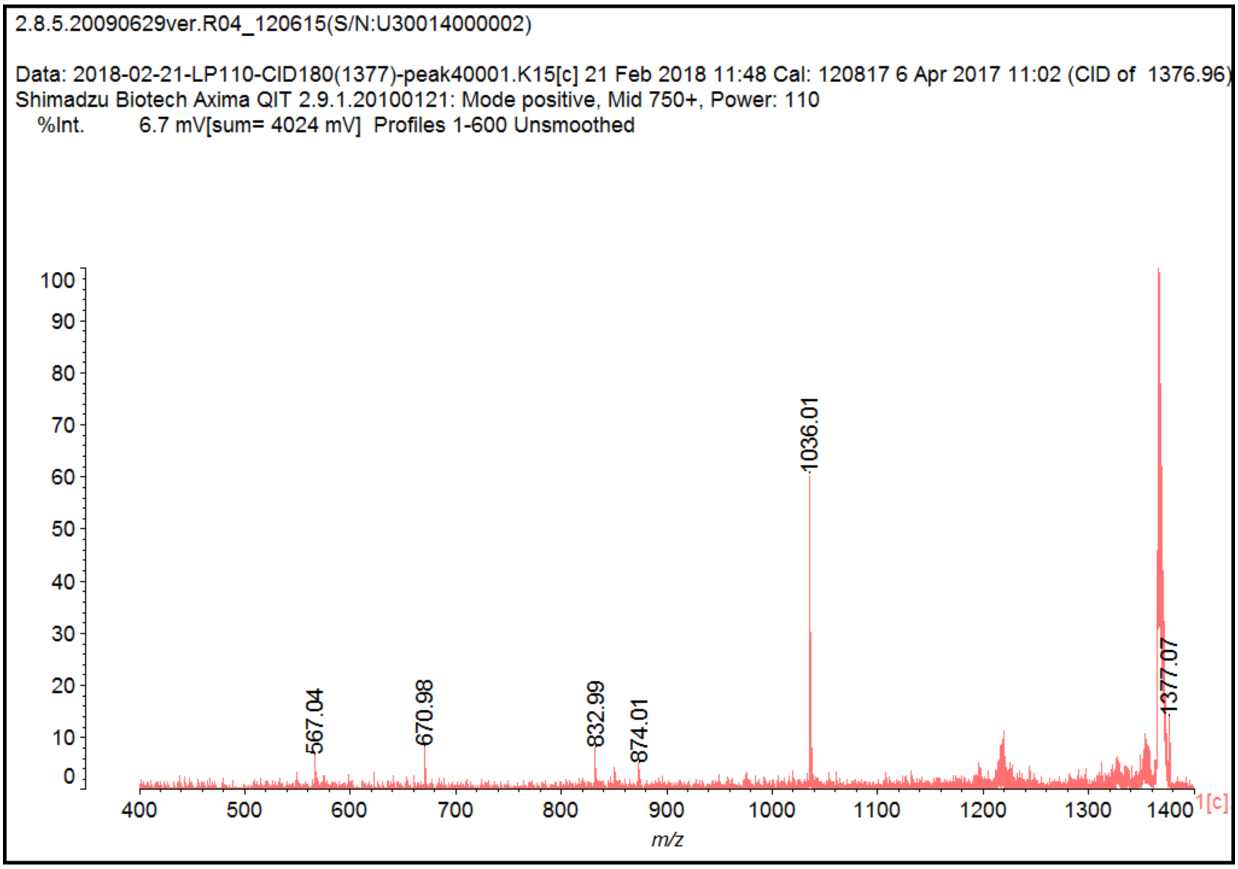

2.8.5.20090629ver.R04_120615(S/N:U30014000002)

Data: 2018-02-21-LP110-CID180(1377)-peak40001.K15[c] 21 Feb 2018 11:48 Cal: 12081
Shimadzu Biotech Axima QIT 2.9.1.20100121: Mode positive, Mid 750+, Power: 110

Mass	%Area	%Total	Apex (mV)	Resolution	S / N	Flags
550.03	5.55	3.41	0.22	0.00	0.00	M
567.04	7.54	4.64	0.42	0.00	0.00	M
670.98	10.93	6.73	0.53	0.00	0.00	M
832.99	9.37	5.77	0.52	0.00	0.00	M
851.01	3.85	2.37	0.27	0.00	0.00	M
874.01	5.65	3.48	0.31	0.00	0.00	M
1036.01	100.00	61.56	4.04	0.00	0.00	M
1377.07	19.55	12.04	0.93	0.00	0.00	M
