# Supplementary material for: Rapid chemical de-N-glycosylation and derivatization for liquid chromatography of immunoglobulin N-linked glycans
Source: PLoS One. 2018 May 3;13(5):e0196800. doi: 10.1371/journal.pone.0196800 (PMC5933716; doi:10.1371/journal.pone.0196800)
Supplement: S6 Fig — (A) MS spectrum, (B) MS/MS spectrum. (PDF) [file pone.0196800.s006.pdf]

A

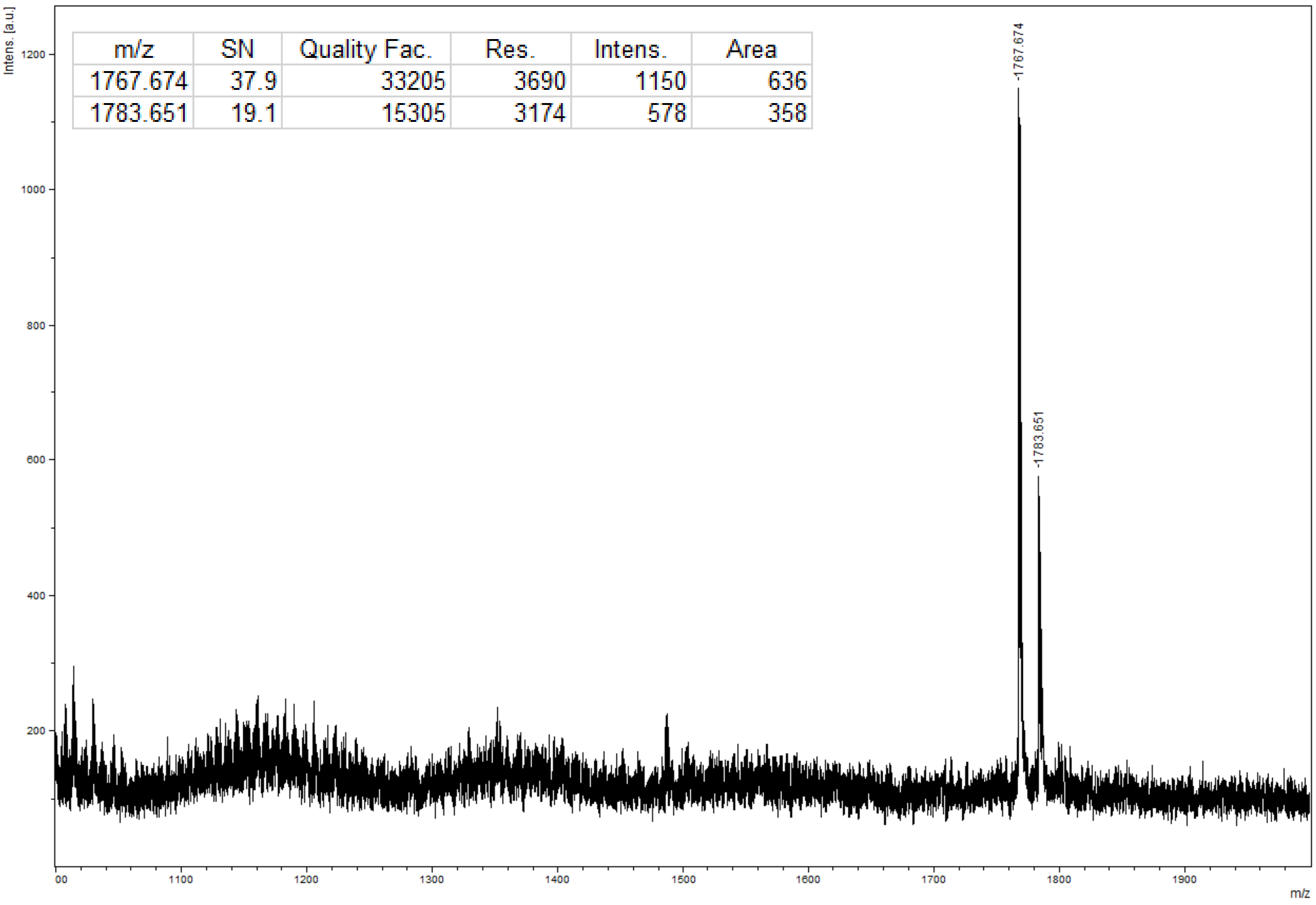

m/z	SN	Quality Fac.	Res.	Intens.	Area
1767.674	37.9	33205	3690	1150	636
1783.651	19.1	15305	3174	578	358

B

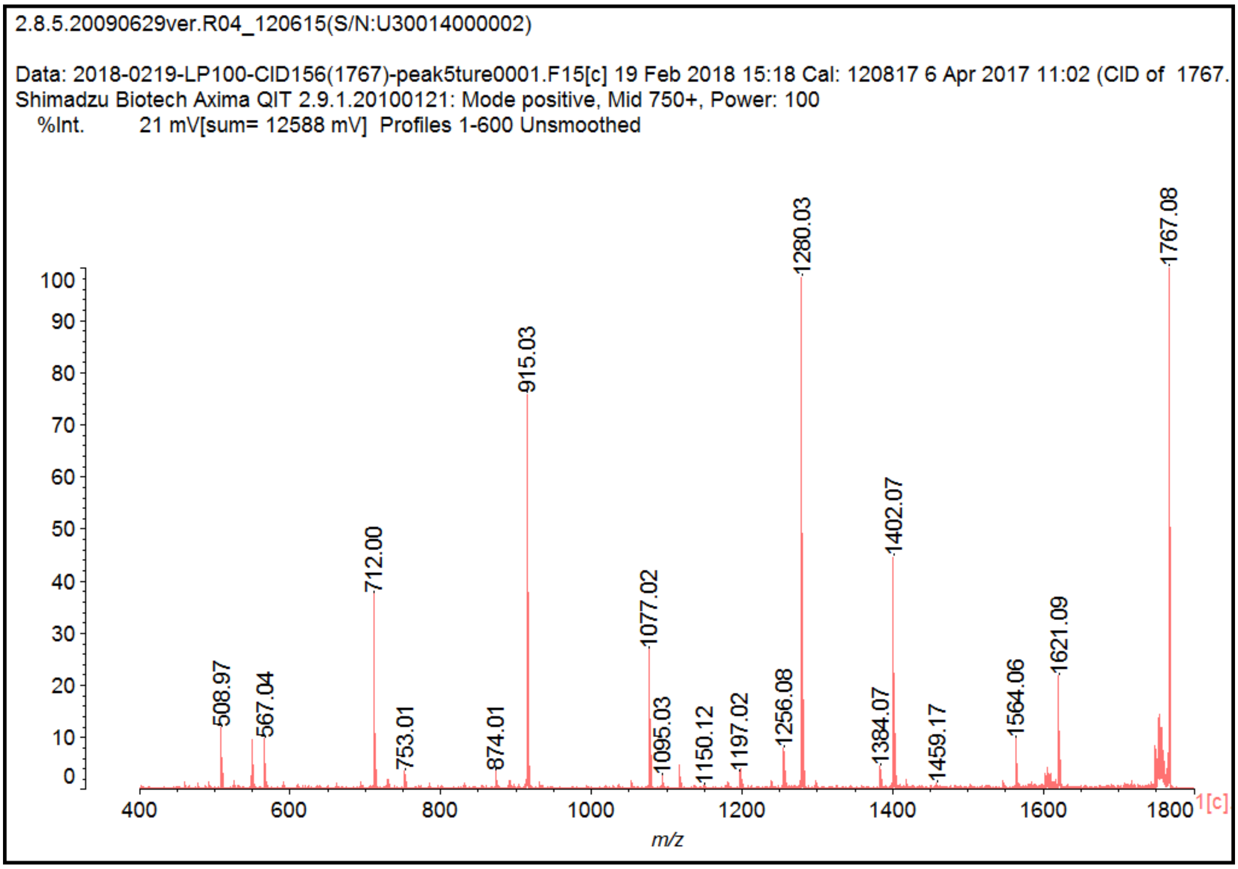

2.8.5.20090629ver.R04_120615(S/N:U30014000002)

Data: 2018-0219-LP100-CID156(1767)-peak5ture0001.F15[c] 19 Feb 2018 15:18 Cal: 12[illegible]
Shimadzu Biotech Axima QIT 2.9.1.20100121: Mode positive, Mid 750+, Power: 100

Mass	%Area	%Total	Apex (mV)	Resolution	S / N	Flags
508.97	9.53	2.03	2.44	0.00	0.00	M
549.99	7.77	1.66	1.96	0.00	0.00	M
567.04	7.87	1.68	2.00	0.00	0.00	M
712.00	33.92	7.22	7.86	0.00	0.00	M
730.01	0.64	0.14	0.37	0.00	0.00	M
753.01	2.84	0.60	0.71	0.00	0.00	M
874.01	3.04	0.65	0.72	0.00	0.00	M
915.03	79.20	16.87	15.90	0.00	0.00	M
1077.02	25.17	5.36	5.61	0.00	0.00	M
1095.03	2.31	0.49	0.52	0.00	0.00	M
1118.02	3.74	0.80	0.95	0.00	0.00	M
1197.02	2.66	0.57	0.65	0.00	0.00	M
1256.08	8.63	1.84	1.61	0.00	0.00	M
1280.03	100.00	21.30	20.59	0.00	0.00	M
1384.07	3.35	0.71	0.91	0.00	0.00	M
1402.07	54.00	11.50	9.33	0.00	0.00	M
1418.02	0.93	0.20	0.37	0.00	0.00	M
1564.06	10.81	2.30	2.01	0.00	0.00	M
1621.09	23.21	4.94	4.54	0.00	0.00	M
1767.08	89.89	19.15	20.98	0.00	0.00	M
