# Supplementary material for: Rapid chemical de-N-glycosylation and derivatization for liquid chromatography of immunoglobulin N-linked glycans
Source: PLoS One. 2018 May 3;13(5):e0196800. doi: 10.1371/journal.pone.0196800 (PMC5933716; doi:10.1371/journal.pone.0196800)
Supplement: S7 Fig — (A) MS spectrum, (B) MS/MS spectrum. (PDF) [file pone.0196800.s007.pdf]

A

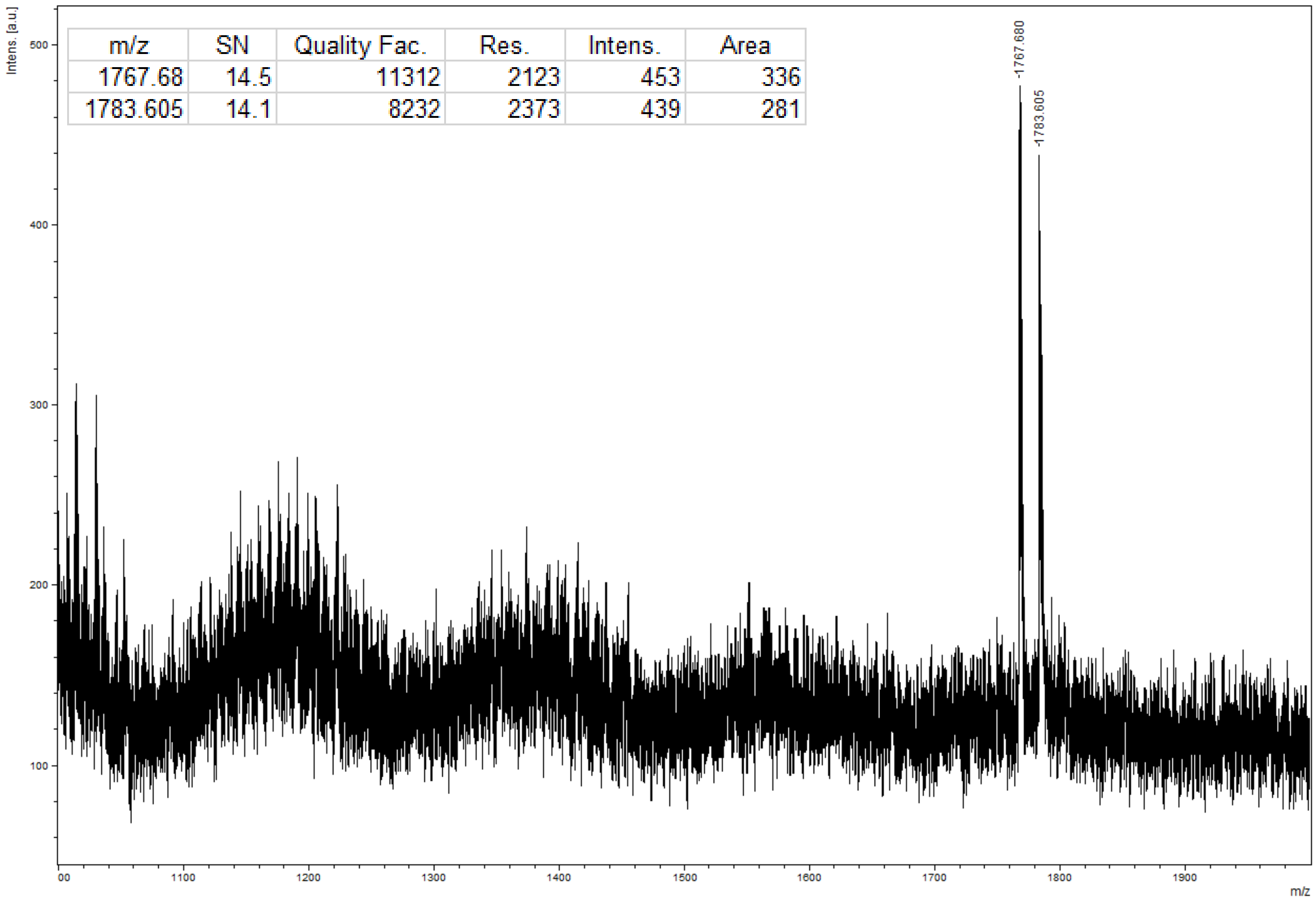

m/z SN Quality Fac. Res. Intens. Area
1767.68 14.5 11312 2123 453 336
1783.605 14.1 8232 2373 439 281
1767.680
1783.605
Intens. [a.u.]
500
400
300
200
100
00
1100
1200
1300
1400
1500
1600
1700
1800
1900
m/z

B

2.8.5.20090629ver.R04_120615(S/N:U30014000002)

Data: 2018-0219-LP100-CID156(1767)-peak60001.G17[c] 19 Feb 2018 15:28 Cal: 120817 6 Apr 2017 11:02 (CID of 1767.11)
Shimadzu Biotech Axima QIT 2.9.1.20100121: Mode positive, Mid 750+, Power: 100
%Int. 3.7 mV[sum= 2212 mV] Profiles 1-600 Unsmoothed

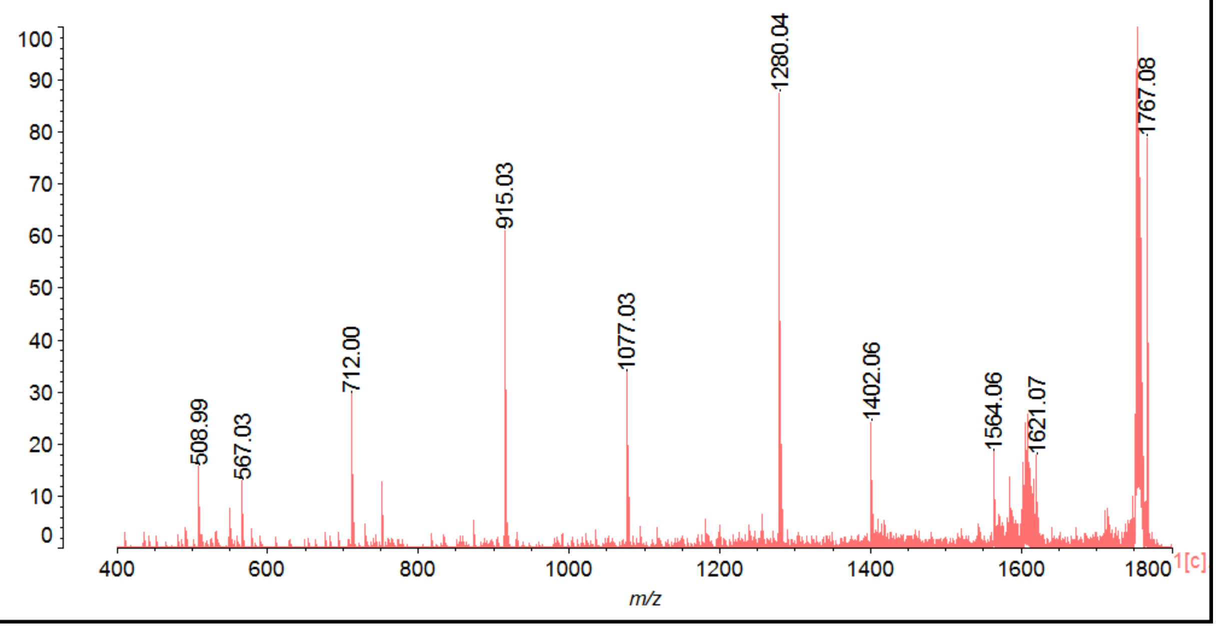

2.8.5.20090629ver.R04_120615(S/N:U30014000002)

Data: 2018-0219-LP100-CID156(1767)-peak60001.G17[c] 19 Feb 2018 15:28 Cal: 12081
Shimadzu Biotech Axima QIT 2.9.1.20100121: Mode positive, Mid 750+, Power: 100

Mass	%Area	%Total	Apex (mV)	Resolution	S / N	Flags
508.99	8.97	2.36	0.58	0.00	0.00	M
550.00	4.74	1.25	0.27	0.00	0.00	M
567.03	9.60	2.52	0.48	0.00	0.00	M
712.00	24.68	6.49	1.09	0.00	0.00	M
753.02	7.79	2.05	0.46	0.00	0.00	M
915.03	45.52	11.97	2.25	0.00	0.00	M
1077.03	37.25	9.79	1.24	0.00	0.00	M
1280.04	70.87	18.63	3.22	0.00	0.00	M
1402.06	22.70	5.97	0.89	0.00	0.00	M
1564.06	16.77	4.41	0.68	0.00	0.00	M
1585.29	8.44	2.22	0.50	0.00	0.00	M
1621.07	23.10	6.07	0.65	0.00	0.00	M
1767.08	100.00	26.29	2.90	0.00	0.00	M
