# Supplementary material for: Rapid chemical de-N-glycosylation and derivatization for liquid chromatography of immunoglobulin N-linked glycans
Source: PLoS One. 2018 May 3;13(5):e0196800. doi: 10.1371/journal.pone.0196800 (PMC5933716; doi:10.1371/journal.pone.0196800)
Supplement: S8 Fig — (A) MS spectrum, (B) MS/MS spectrum. (PDF) [file pone.0196800.s008.pdf]

A

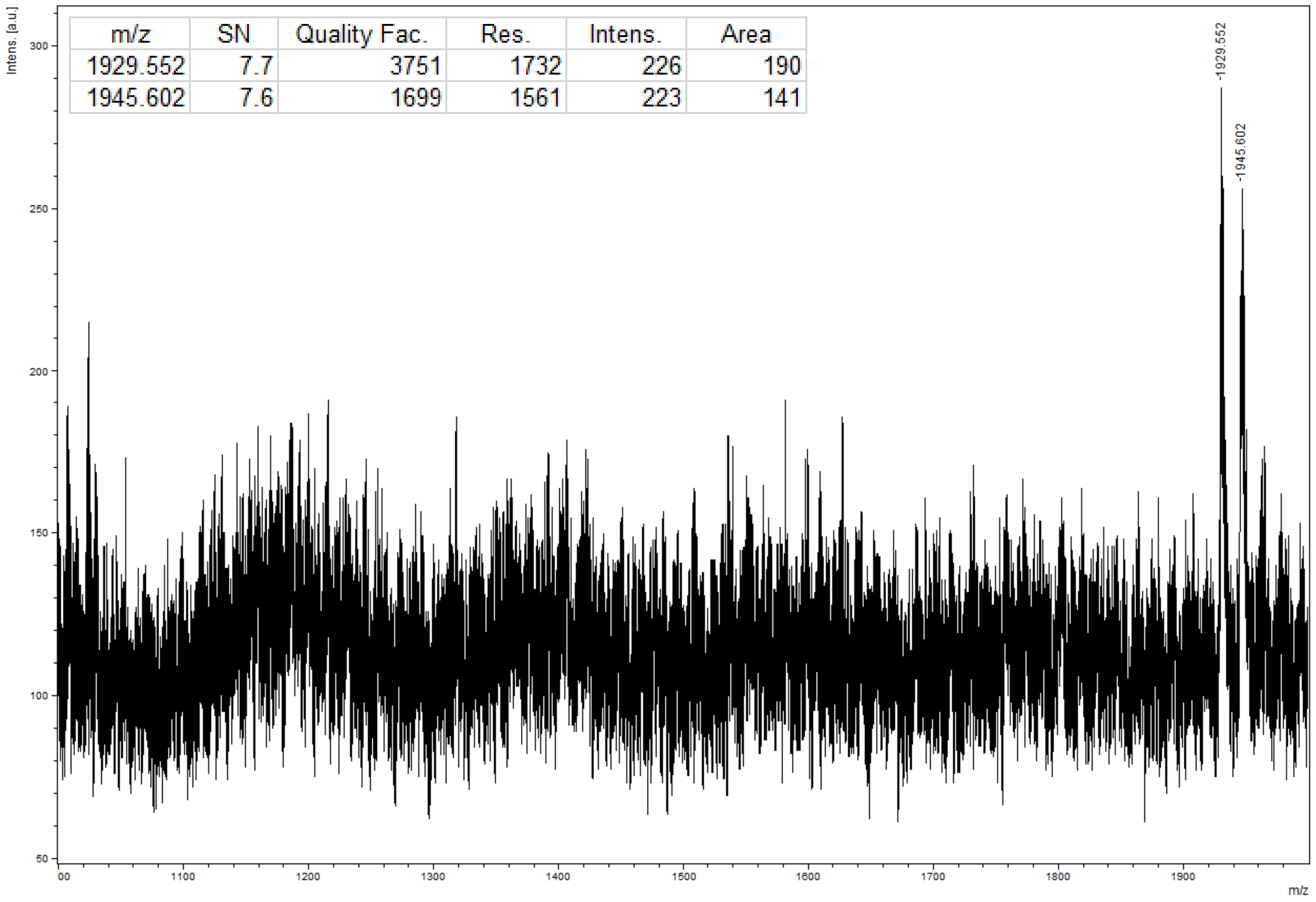

m/z	SN	Quality Fac.	Res.	Intens.	Area
1929.552	7.7	3751	1732	226	190
1945.602	7.6	1699	1561	223	141

B

2.8.5.20090629ver.R04_120615(S/N:U30014000002)

Data: 2018-0219-LP115-CID156(1929)-peak70001.H15[c] 19 Feb 2018 16:15 Cal: 120817 6 Apr 2017 11:02 (CID of 1929.27)
Shimadzu Biotech Axima QIT 2.9.1.20100121: Mode positive, Mid 750+, Power: 115
%Int. 2.9 mV[sum= 1749 mV] Profiles 1-600 Unsmoothed

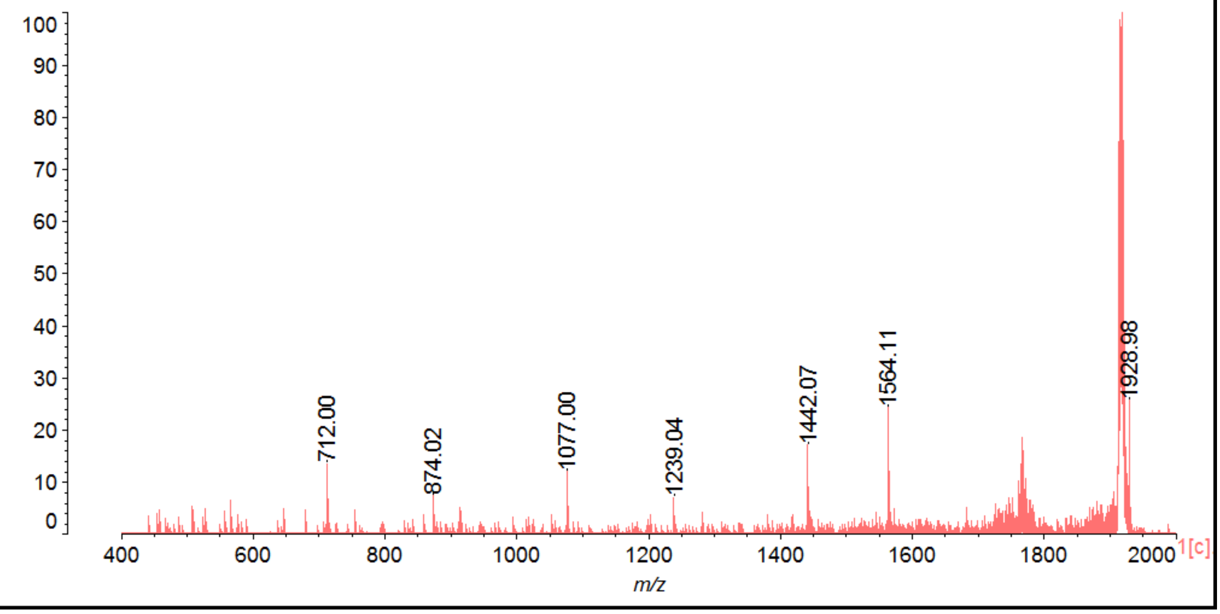

2.8.5.20090629ver.R04_120615(S/N:U30014000002)

Data: 2018-0219-LP115-CID156(1929)-peak70001.H15[c] 19 Feb 2018 16:15 Cal: 12081[illegible]
Shimadzu Biotech Axima QIT 2.9.1.20100121: Mode positive, Mid 750+, Power: 115

Mass	%Area	%Total	Apex (mV)	Resolution	S / N	Flags
712.00	29.73	8.86	0.39	0.00	0.00	M
874.02	22.29	6.64	0.22	0.00	0.00	M
915.04	12.64	3.77	0.14	0.00	0.00	M
1077.00	49.32	14.69	0.35	0.00	0.00	M
1239.04	25.29	7.53	0.20	0.00	0.00	M
1442.07	73.43	21.88	0.50	0.00	0.00	M
1564.11	100.00	29.79	0.71	0.00	0.00	M
1928.98	22.97	6.84	0.75	0.00	0.00	M
