# Supplementary figures and images for: Rapid chemical de-N-glycosylation and derivatization for liquid chromatography of immunoglobulin N-linked glycans
Source: PLoS One. 2018 May 3;13(5):e0196800. doi: 10.1371/journal.pone.0196800 (PMC5933716; doi:10.1371/journal.pone.0196800)

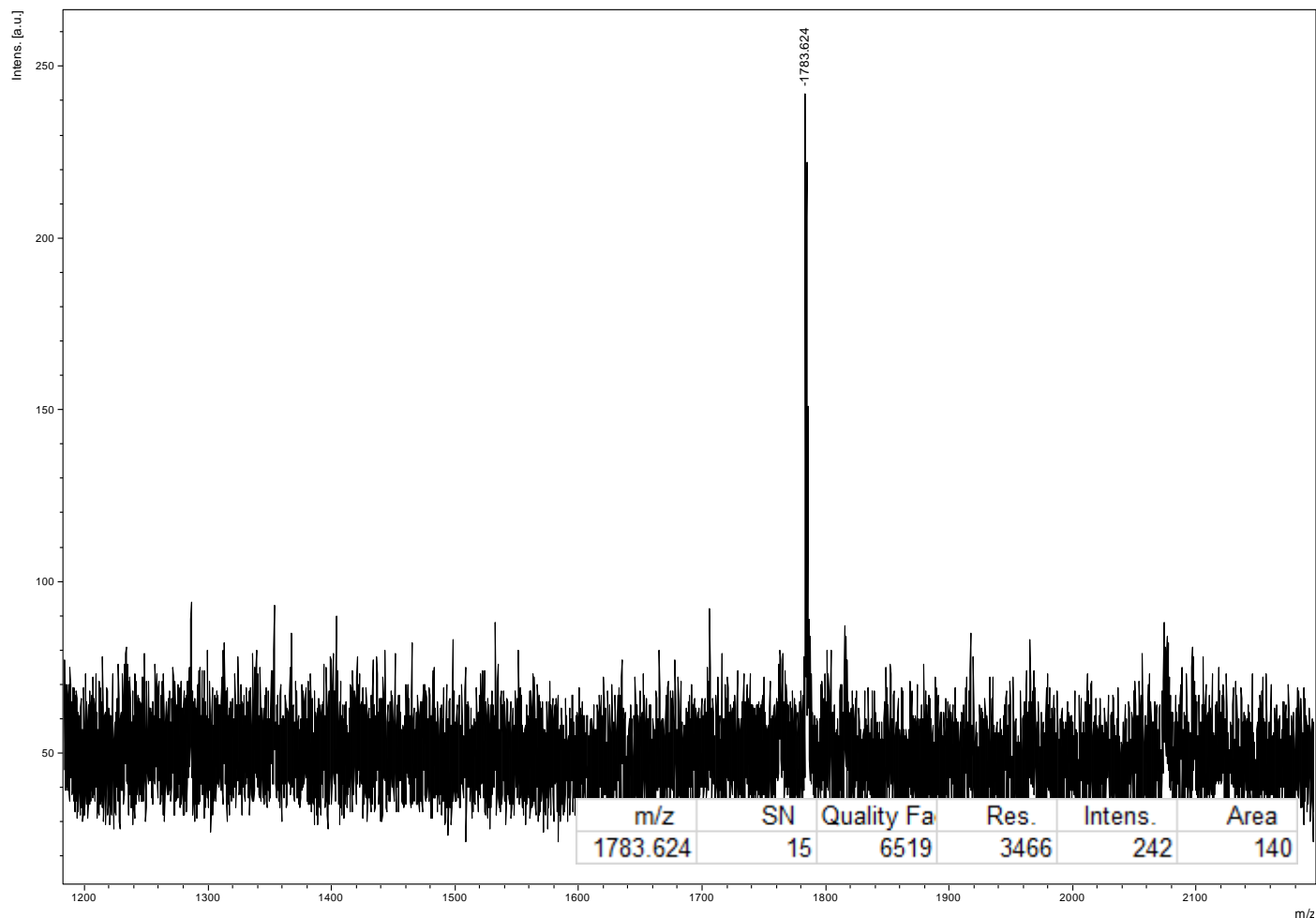

Supplement: S10 Fig — (PDF) [file pone.0196800.s010.pdf]

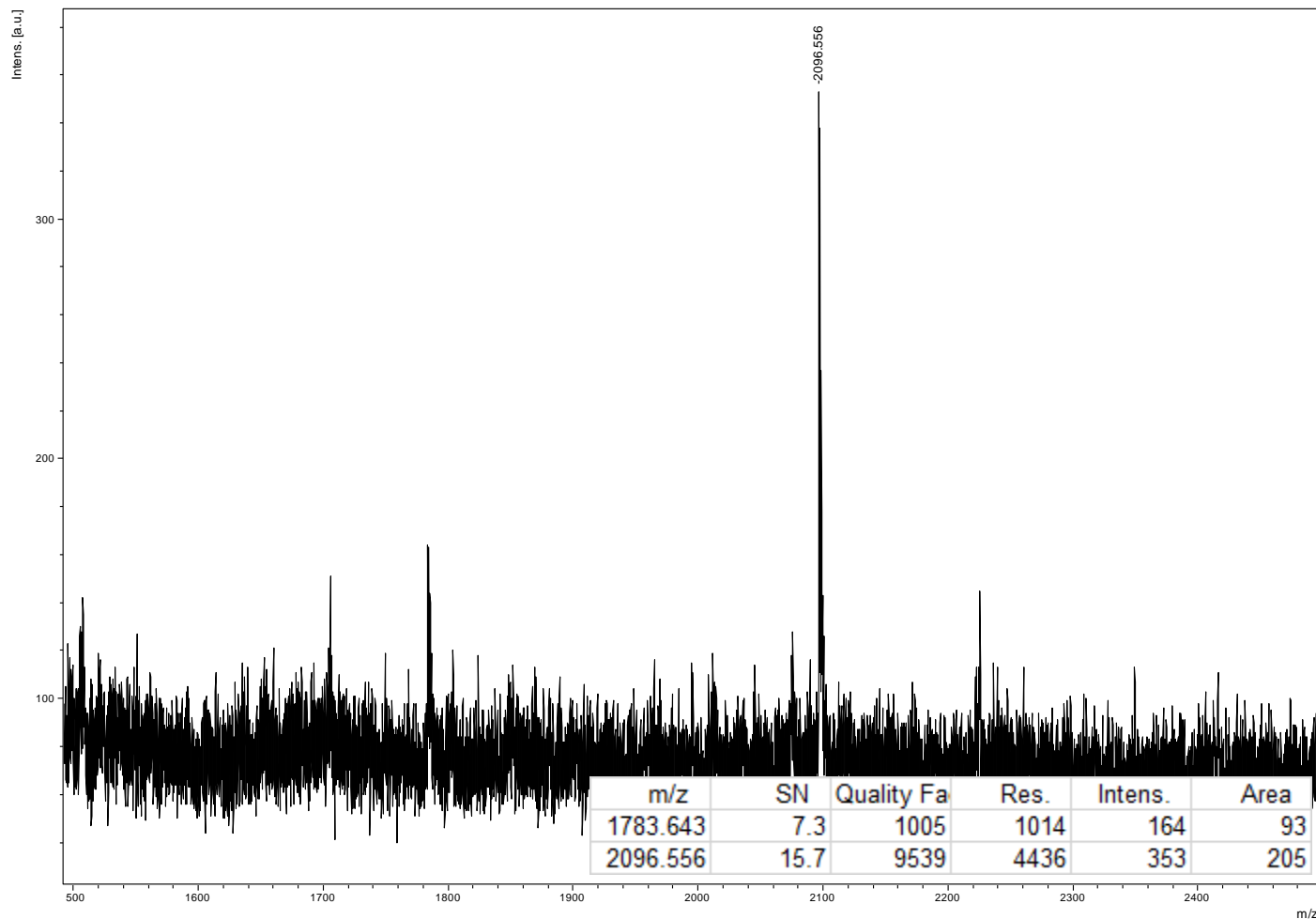

Supplement: S11 Fig — (PDF) [file pone.0196800.s011.pdf]

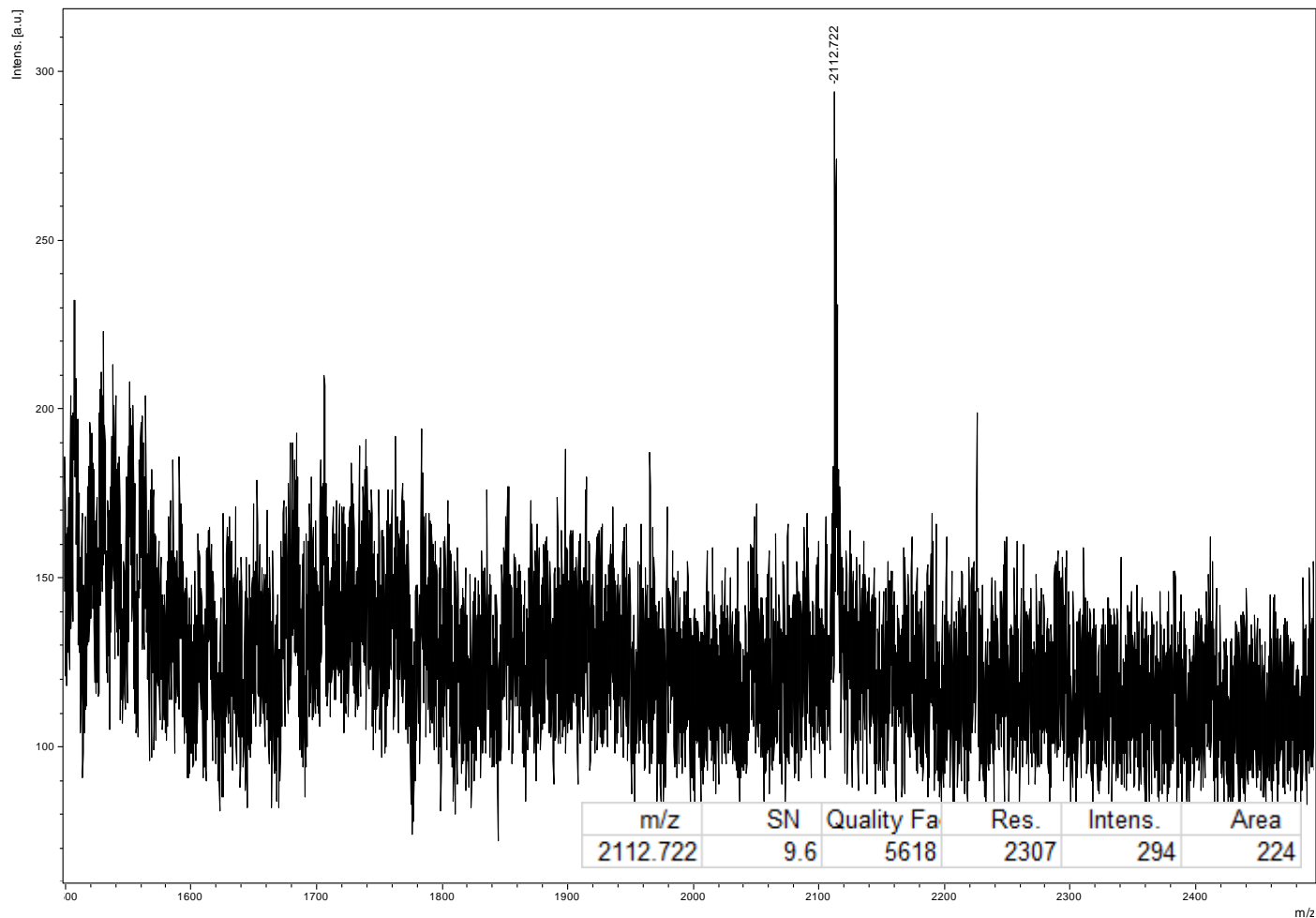

Supplement: S12 Fig — (PDF) [file pone.0196800.s012.pdf]

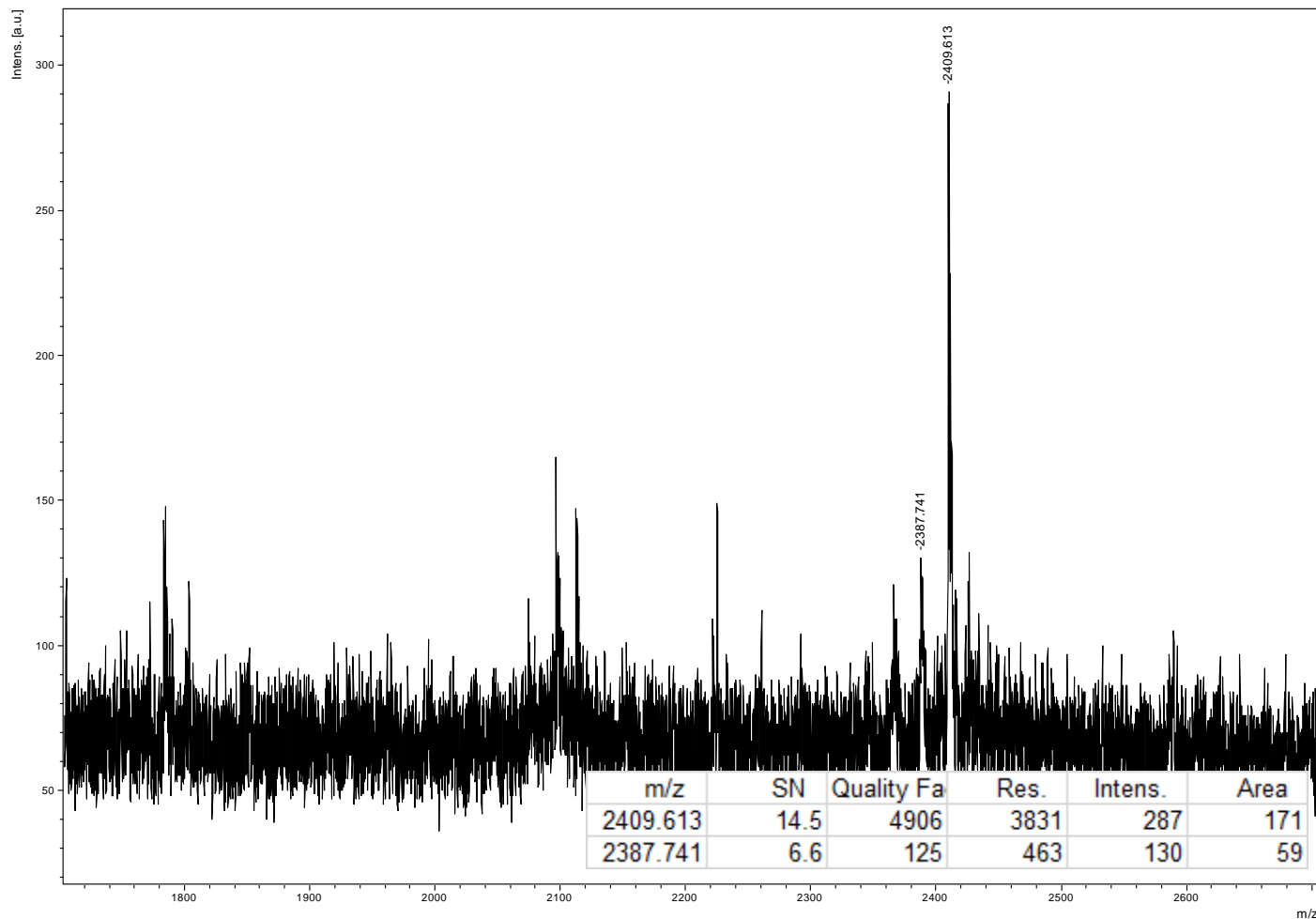

Supplement: S13 Fig — (PDF) [file pone.0196800.s013.pdf]

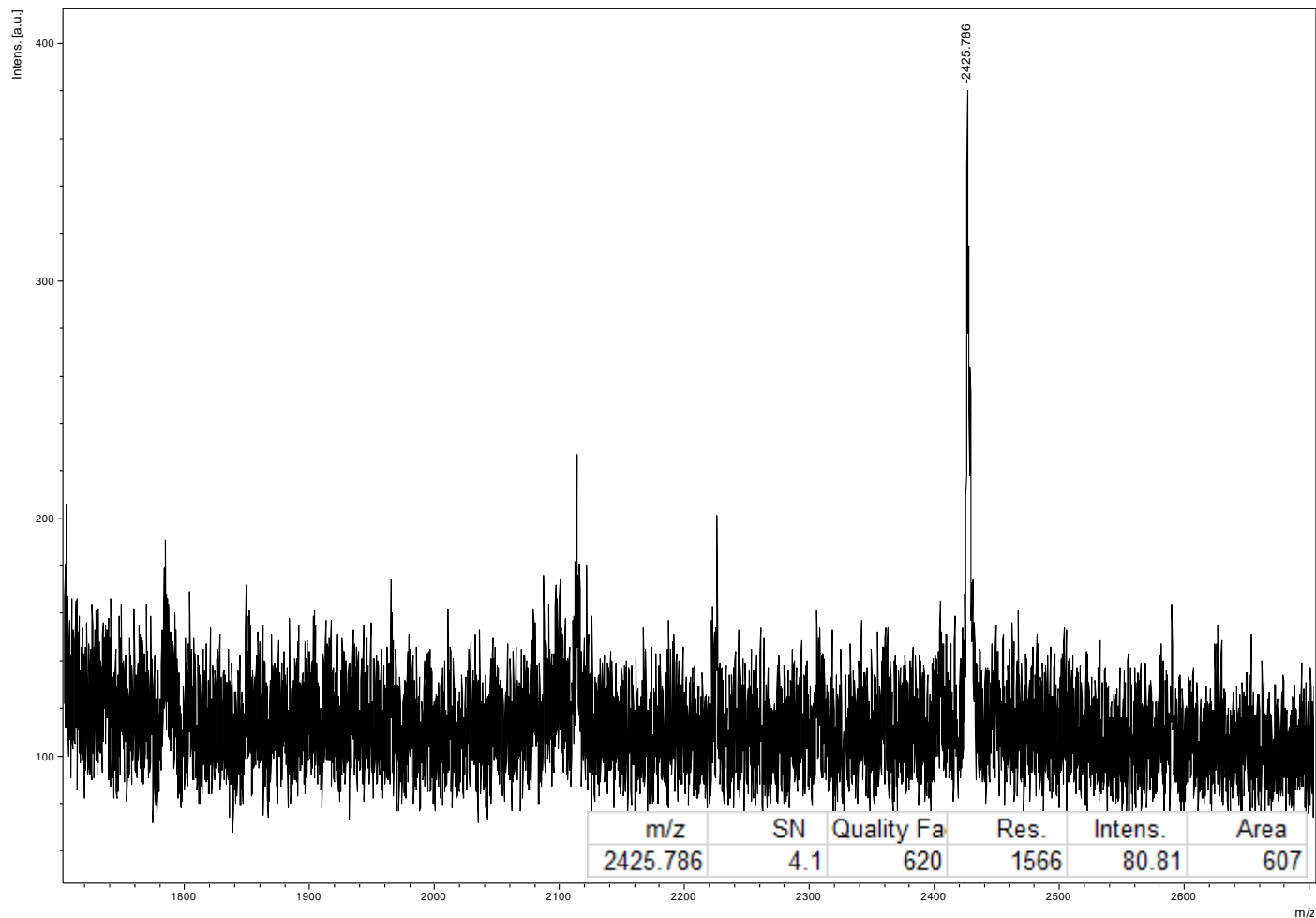

Supplement: S14 Fig — (PDF) [file pone.0196800.s014.pdf]

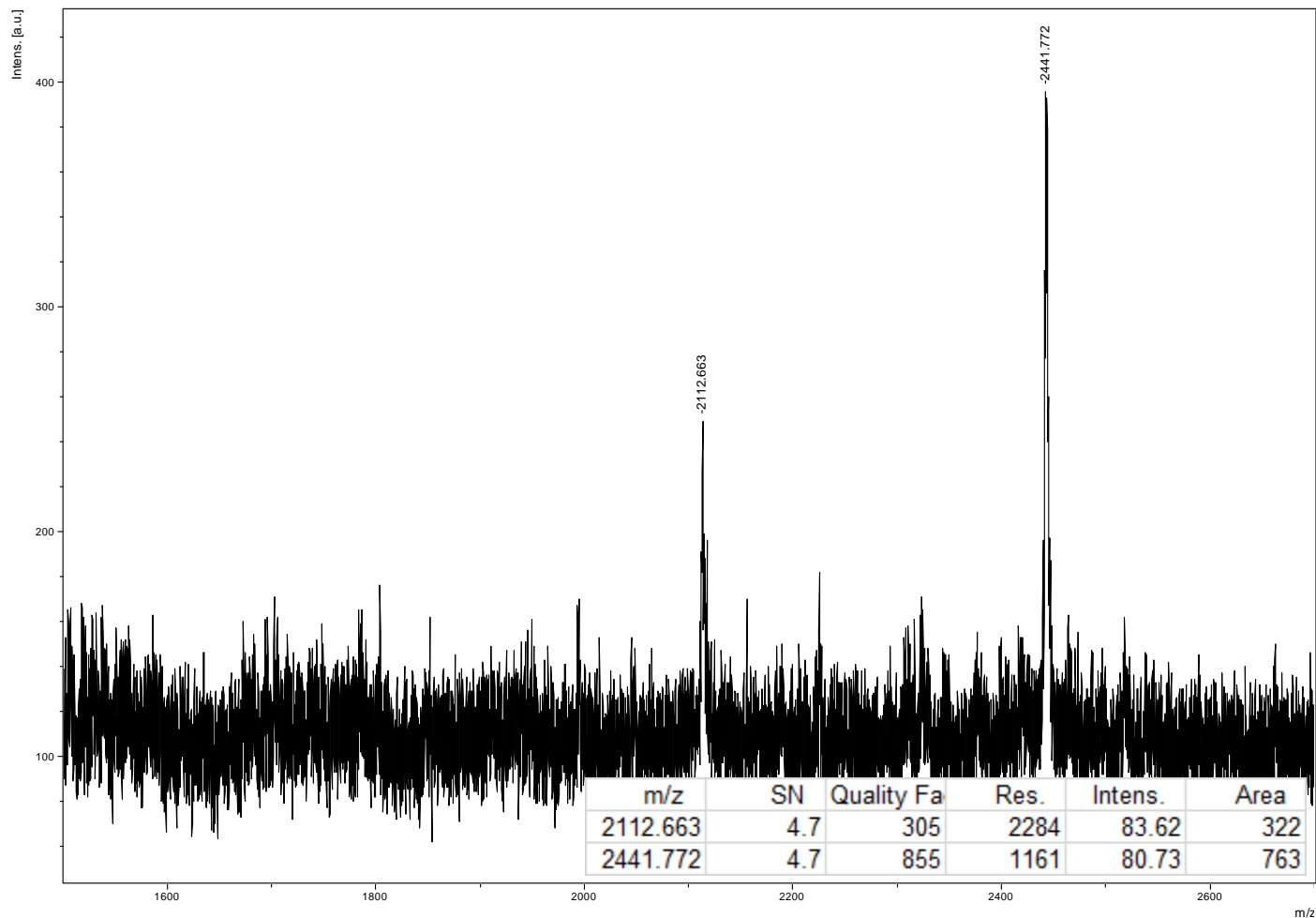

Supplement: S15 Fig — (PDF) [file pone.0196800.s015.pdf]

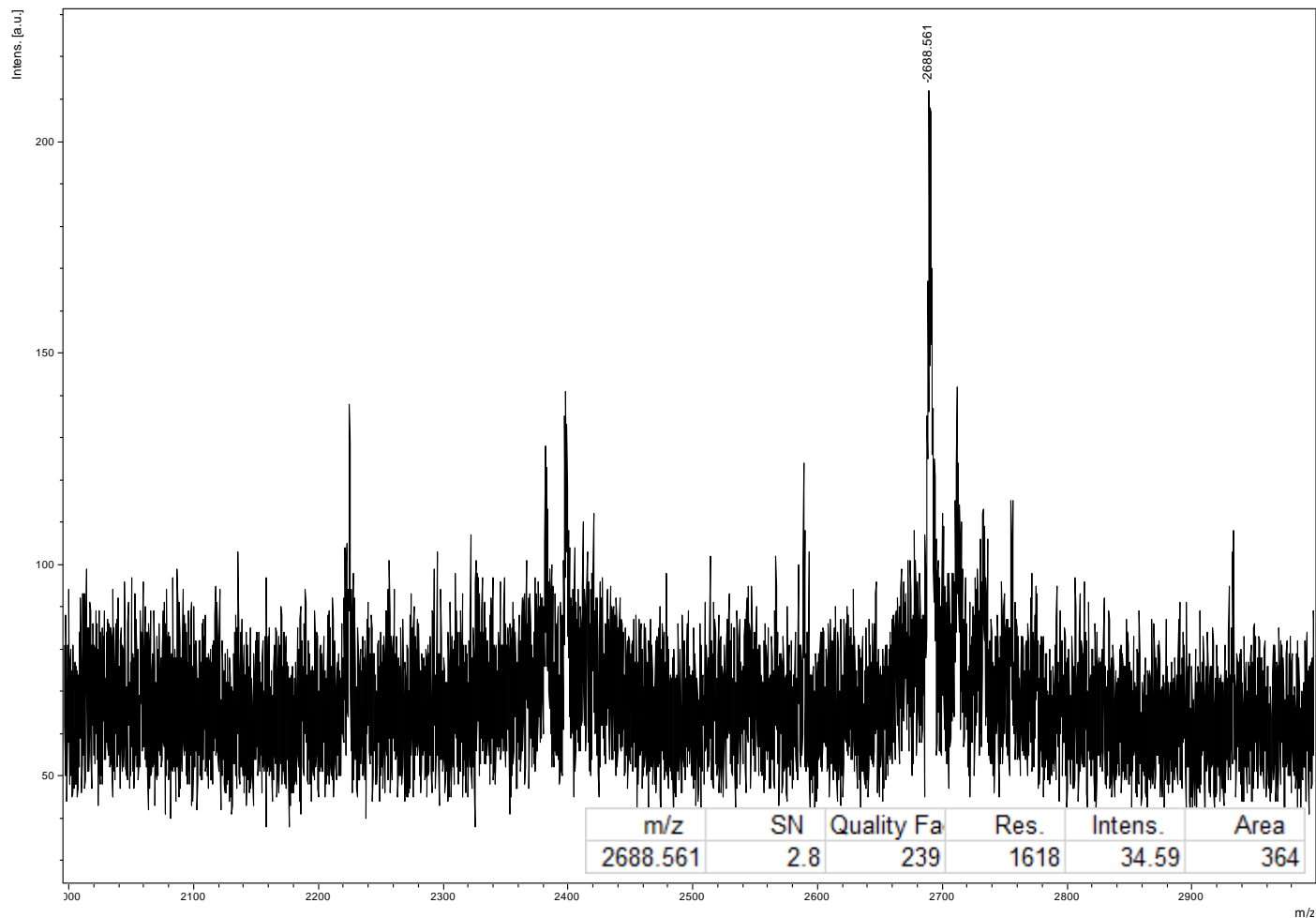

Supplement: S16 Fig — (PDF) [file pone.0196800.s016.pdf]

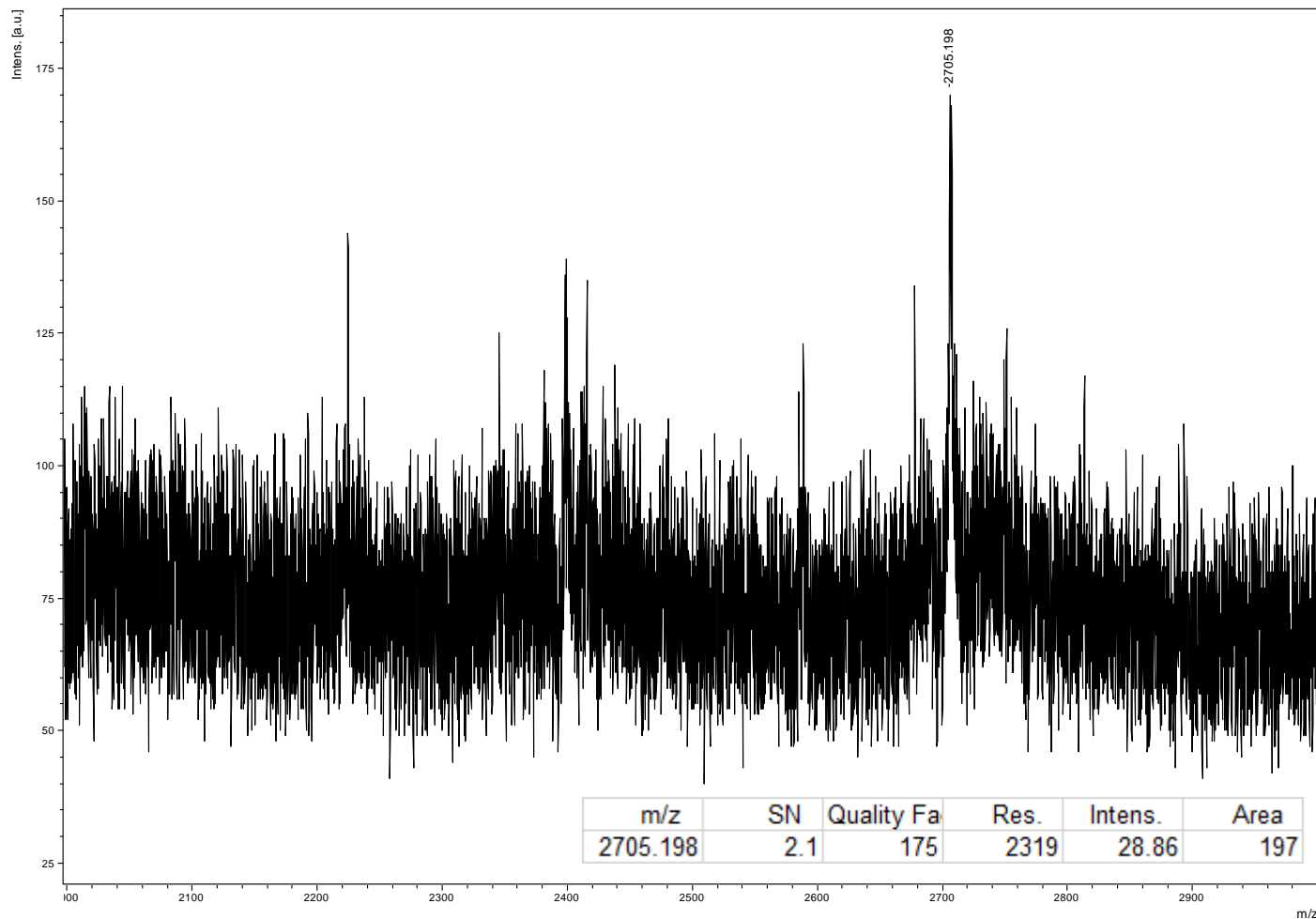

Supplement: S17 Fig — (PDF) [file pone.0196800.s017.pdf]

A

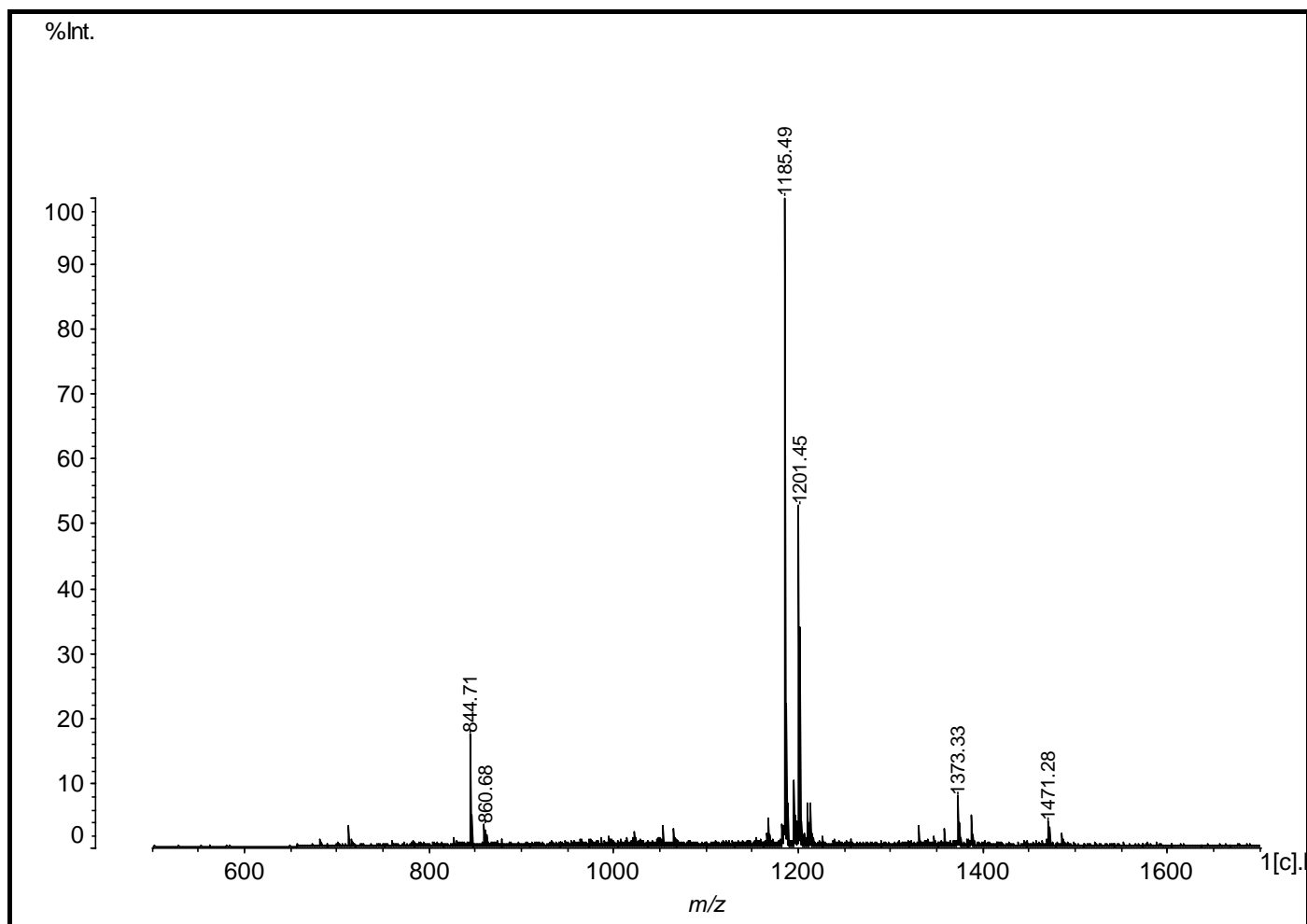

B

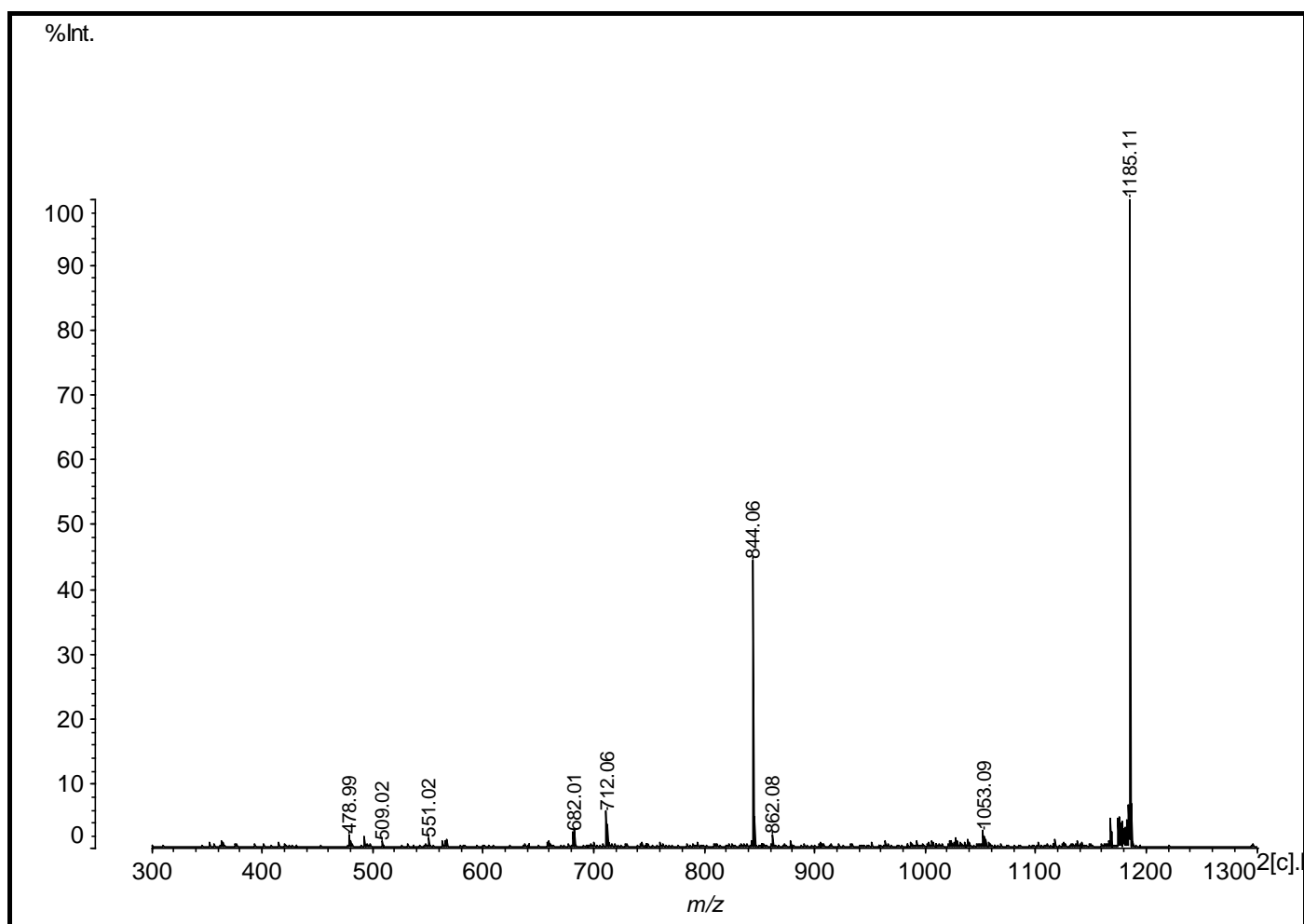

Supplement: S18 Fig — (A) MS spectrum, (B) MS/MS spectrum. (PDF) [file pone.0196800.s018.pdf]

A

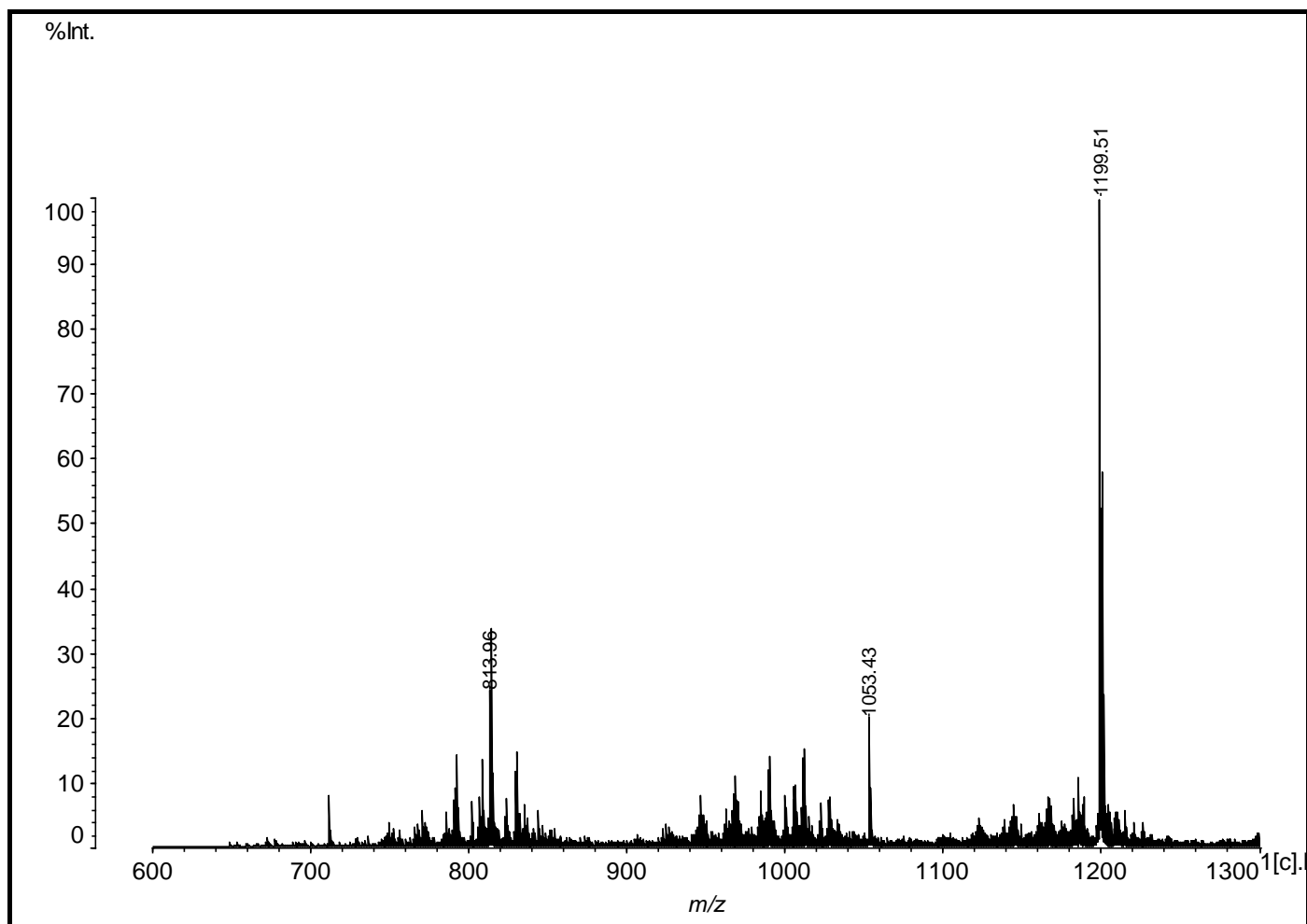

B

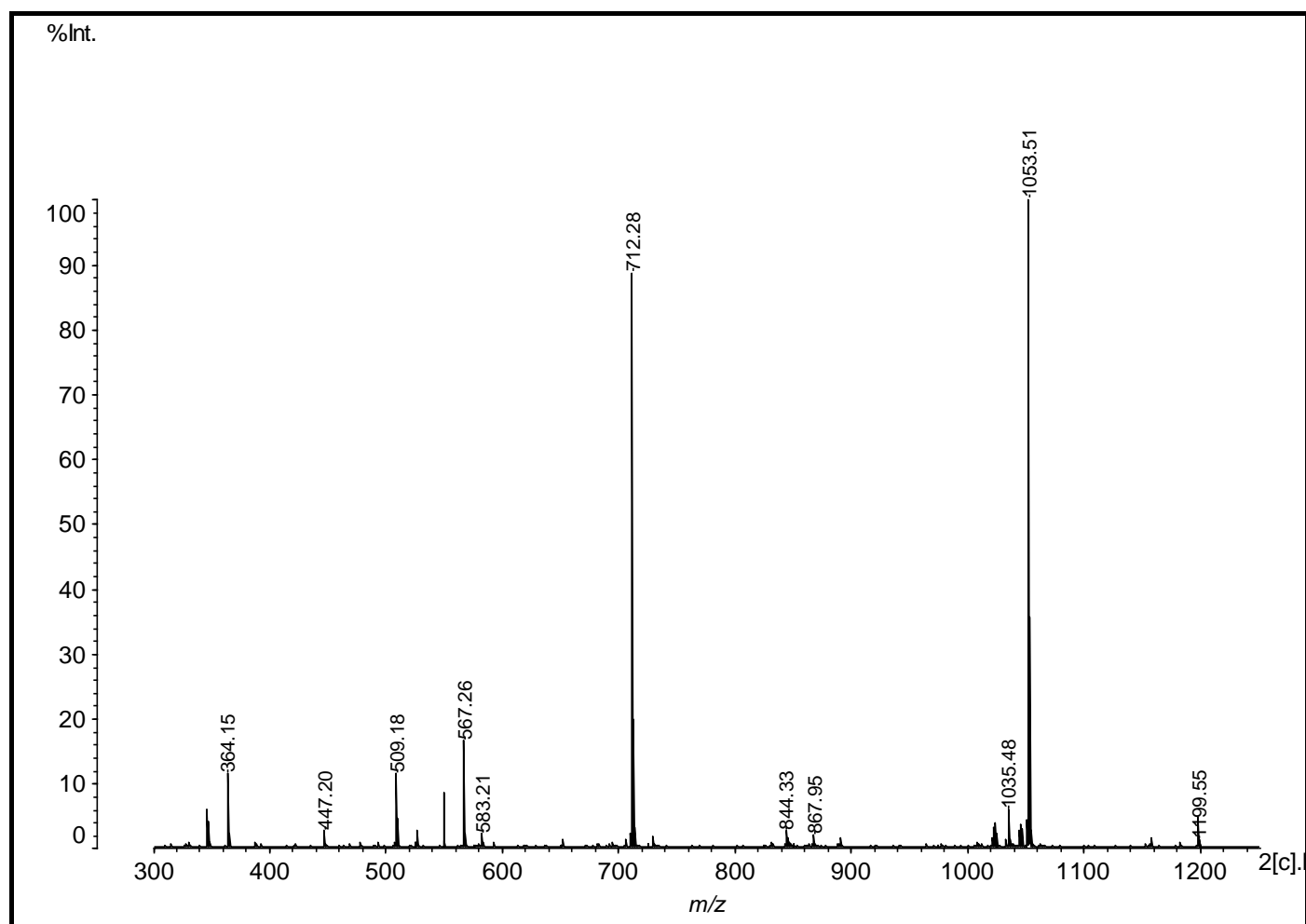

Supplement: S19 Fig — (A) MS spectrum, (B) MS/MS spectrum. (PDF) [file pone.0196800.s019.pdf]

A

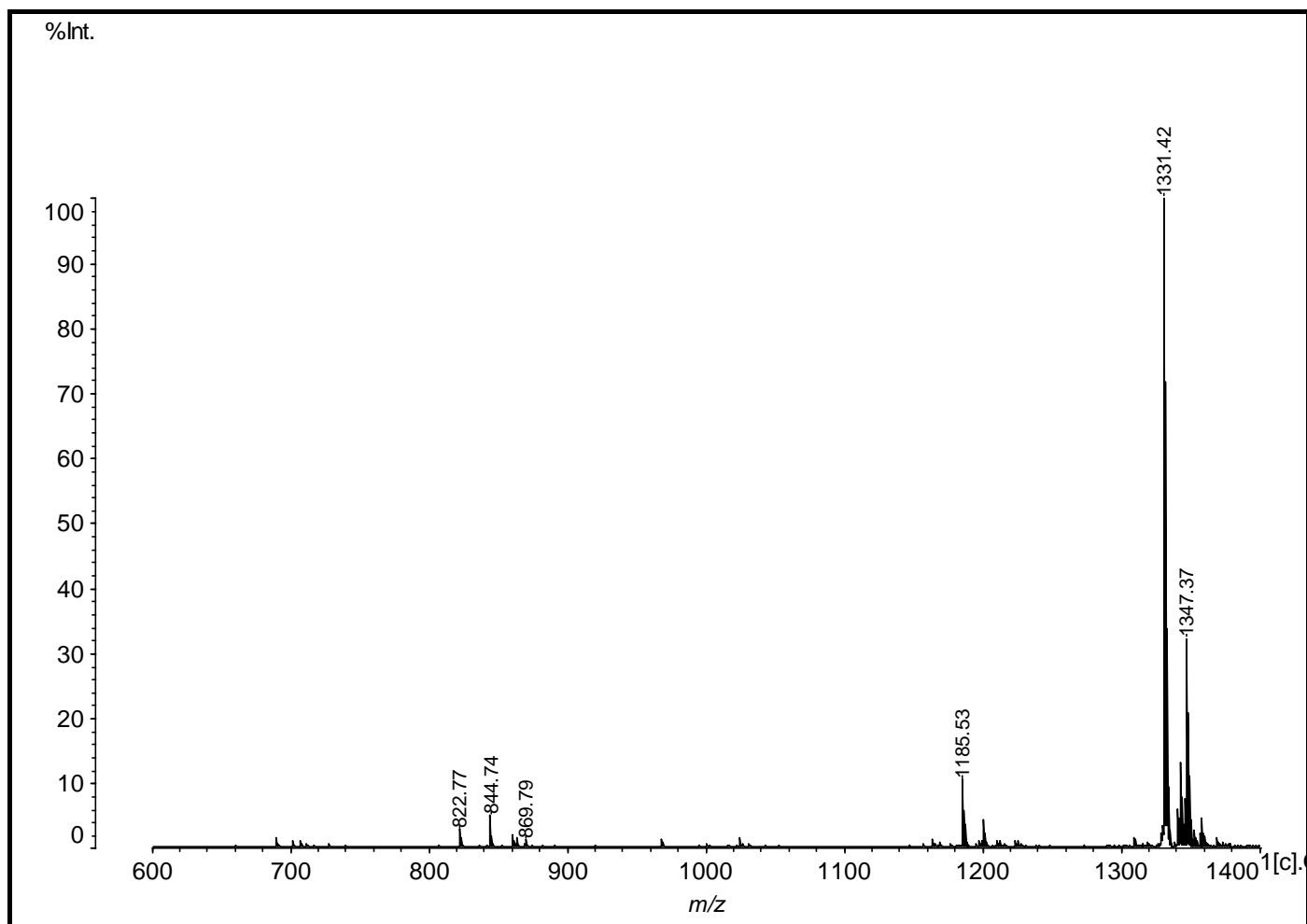

B

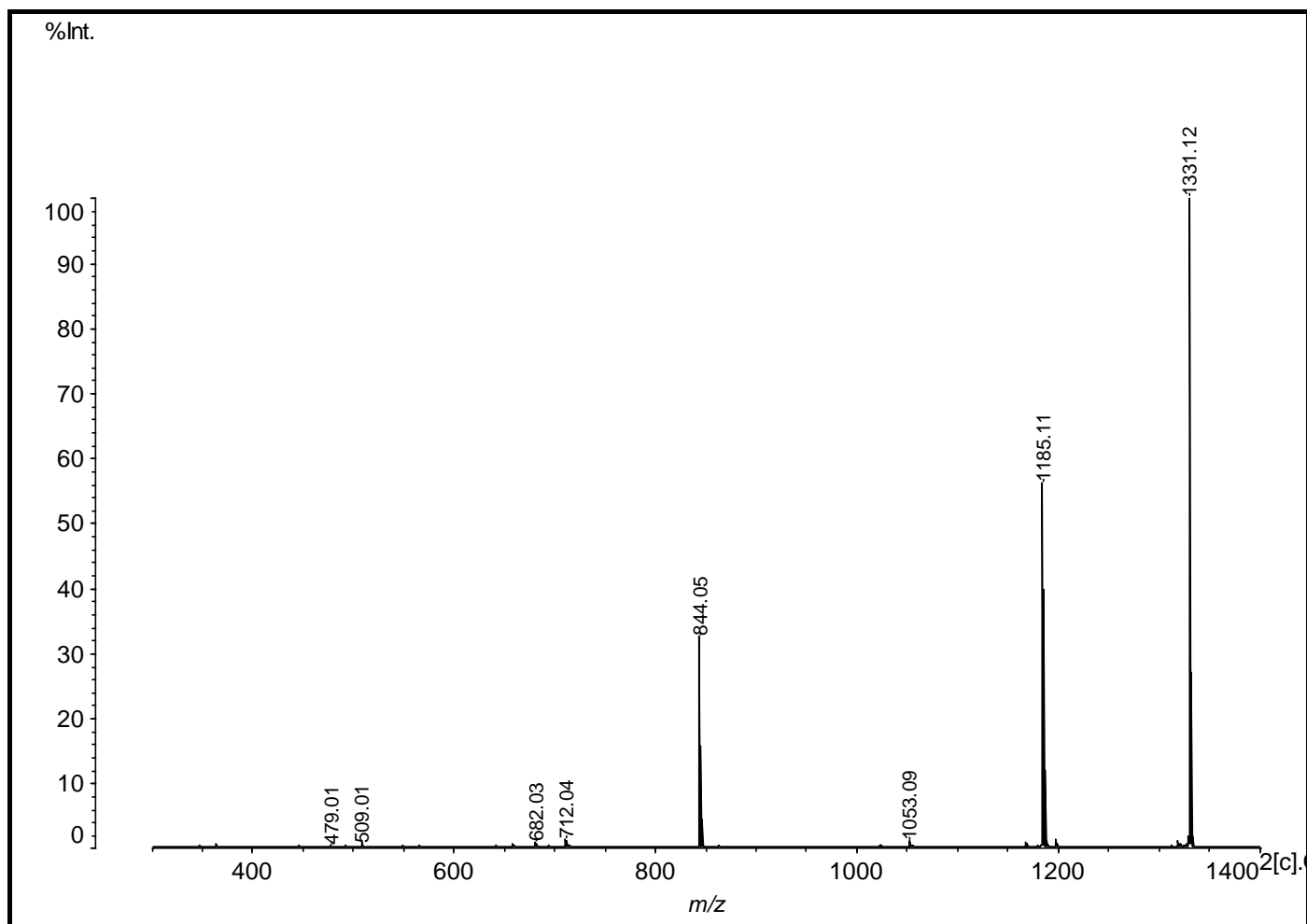

Supplement: S20 Fig — (A) MS spectrum, (B) MS/MS spectrum. (PDF) [file pone.0196800.s020.pdf]

A

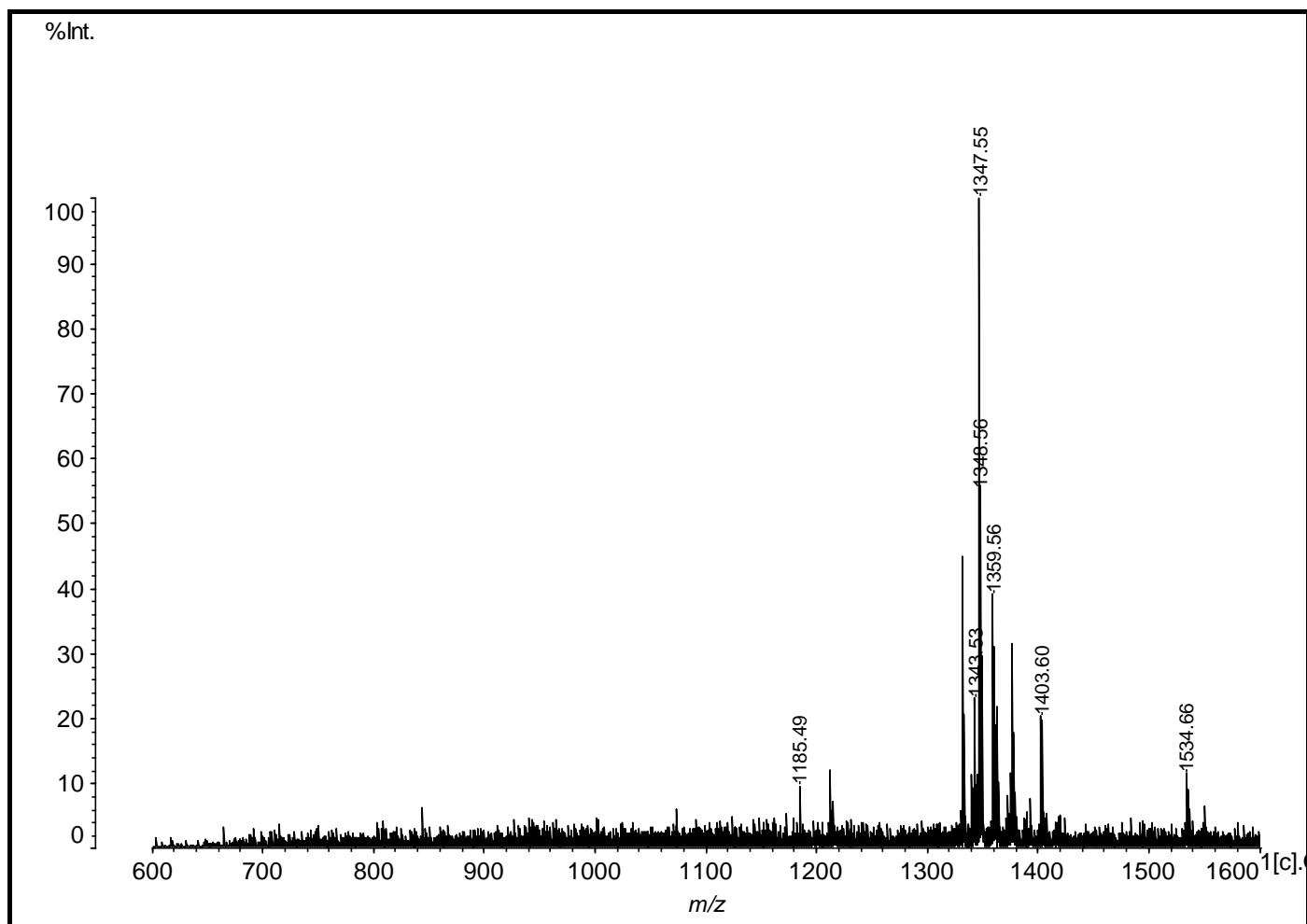

B

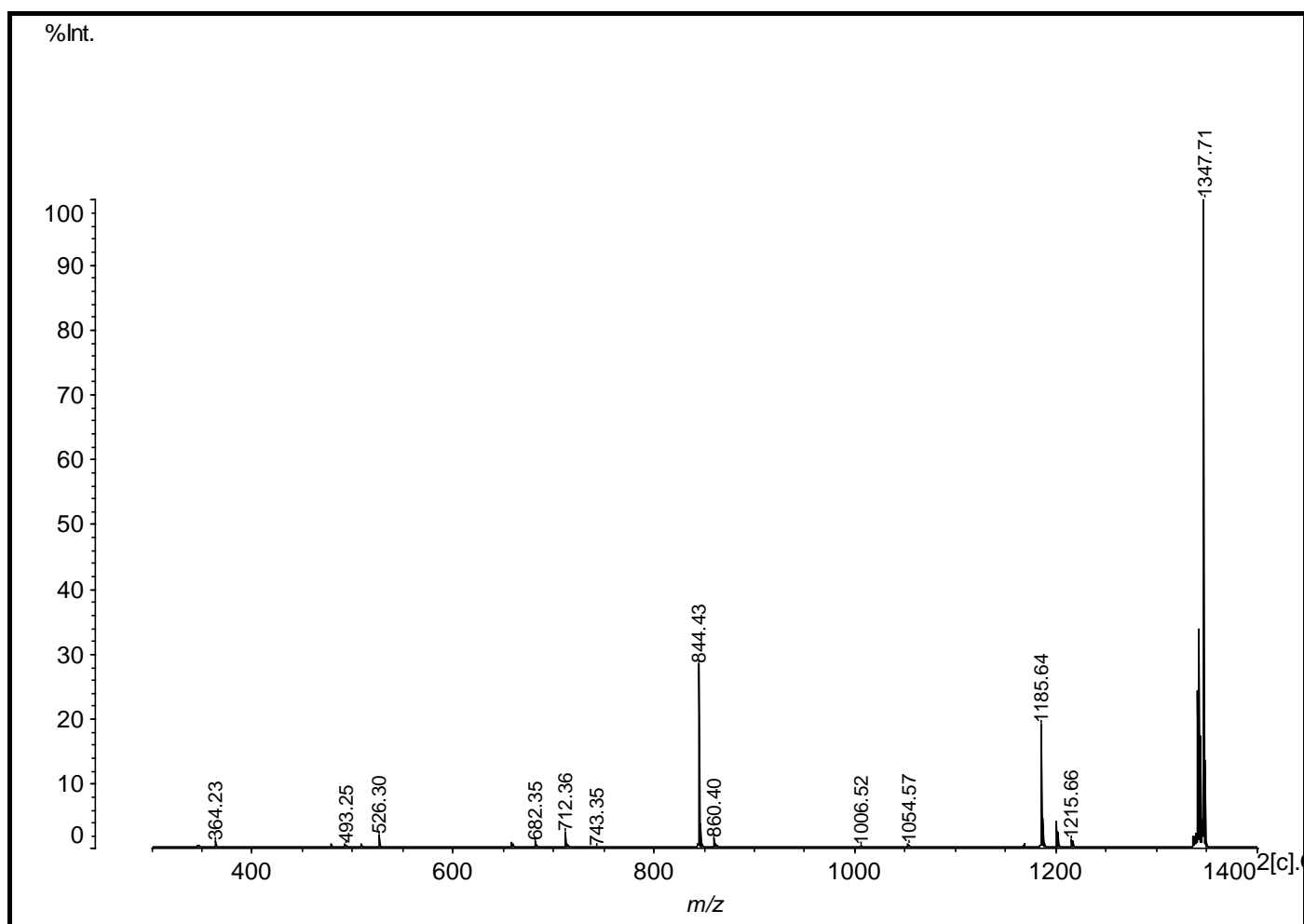

Supplement: S21 Fig — (A) MS spectrum, (B) MS/MS spectrum. (PDF) [file pone.0196800.s021.pdf]

A

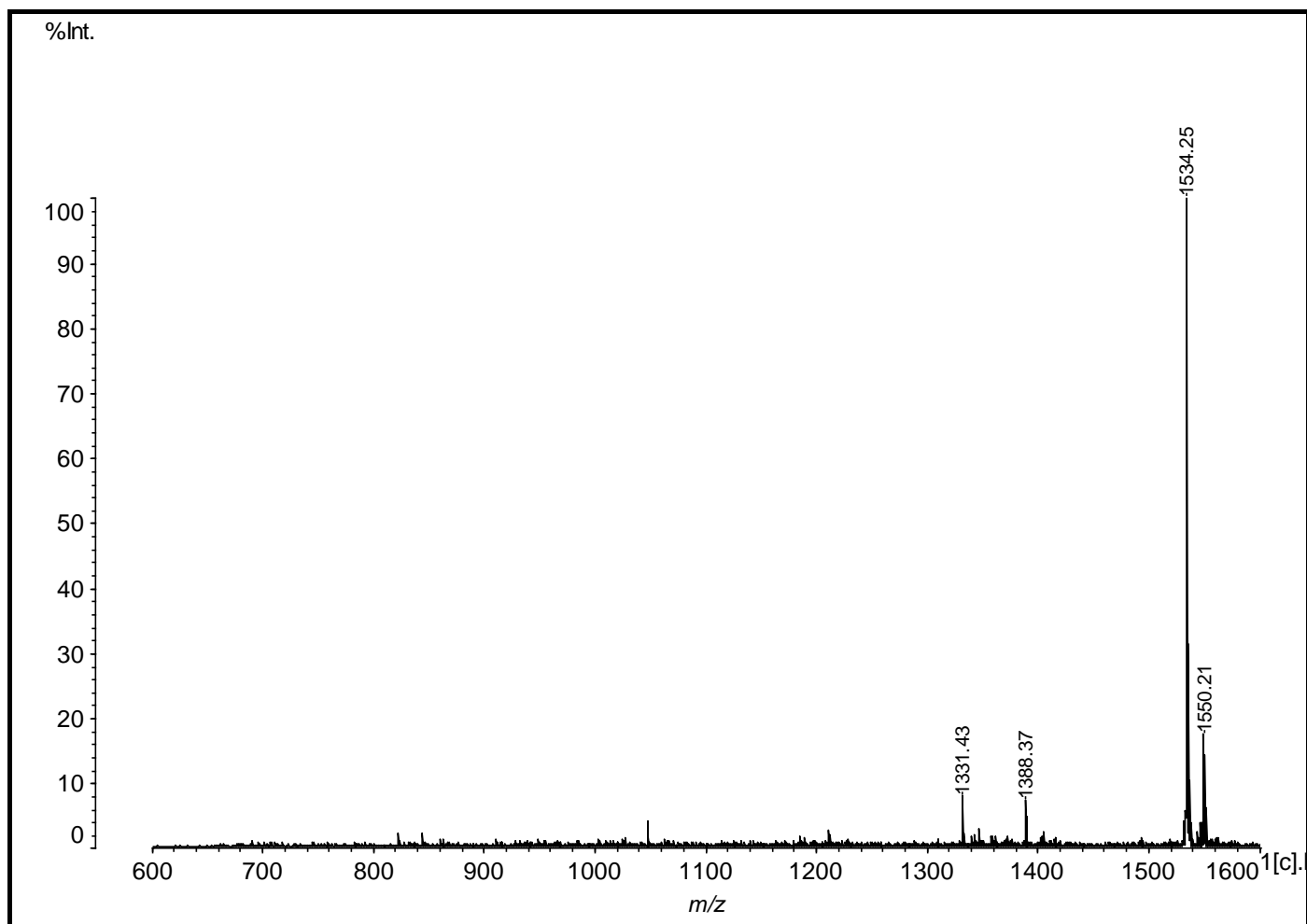

B

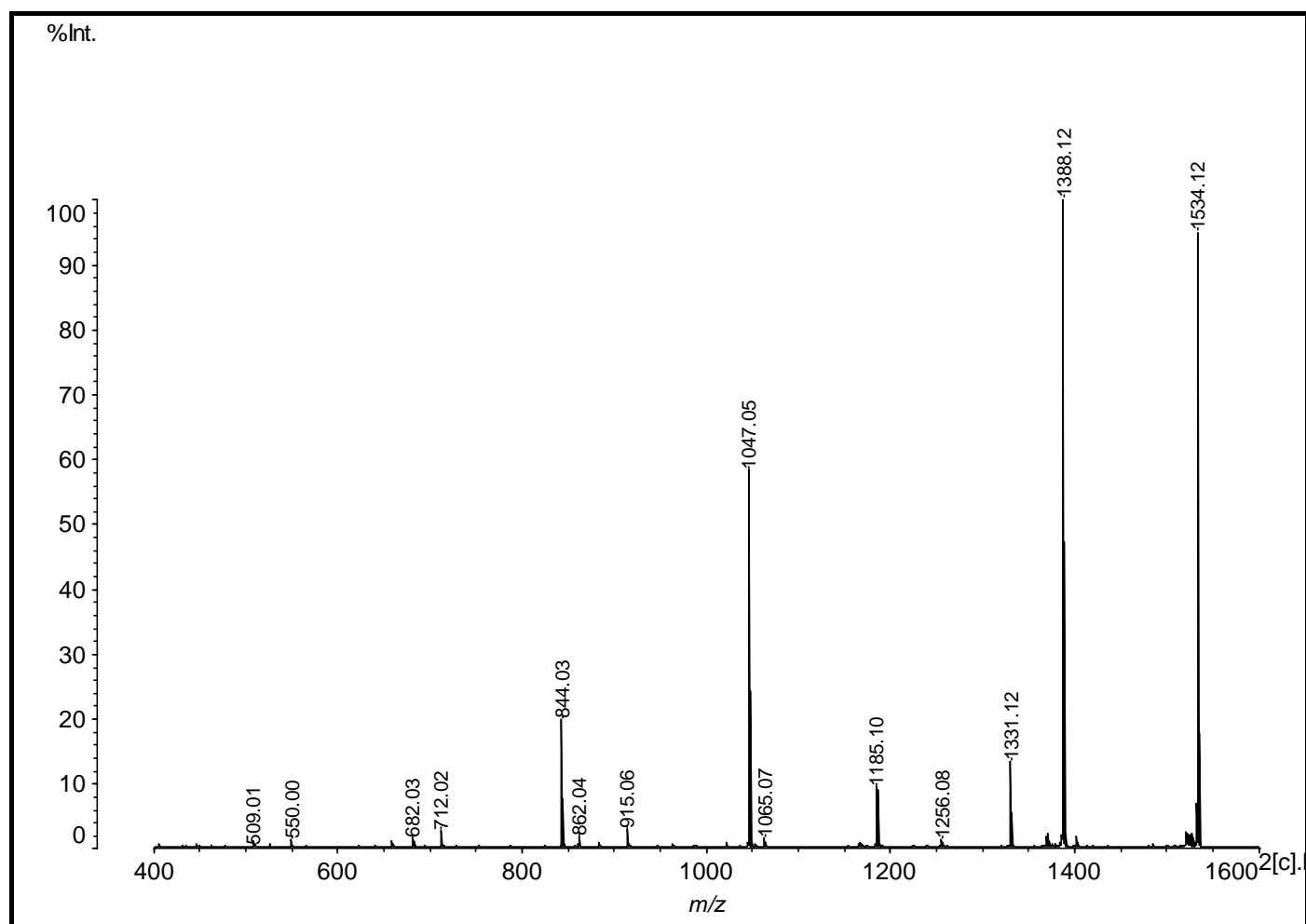

Supplement: S22 Fig — (A) MS spectrum, (B) MS/MS spectrum. (PDF) [file pone.0196800.s022.pdf]

A

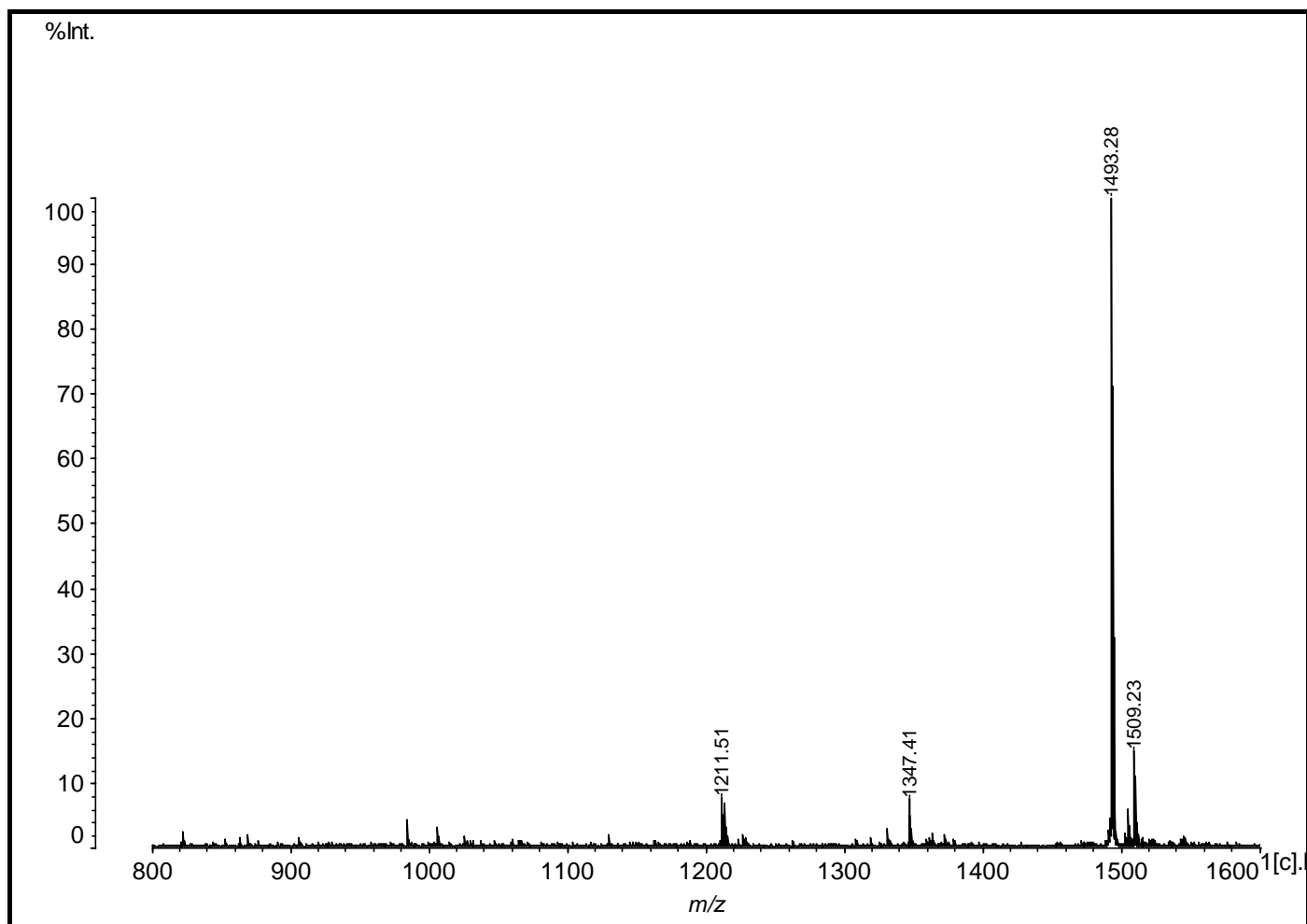

B

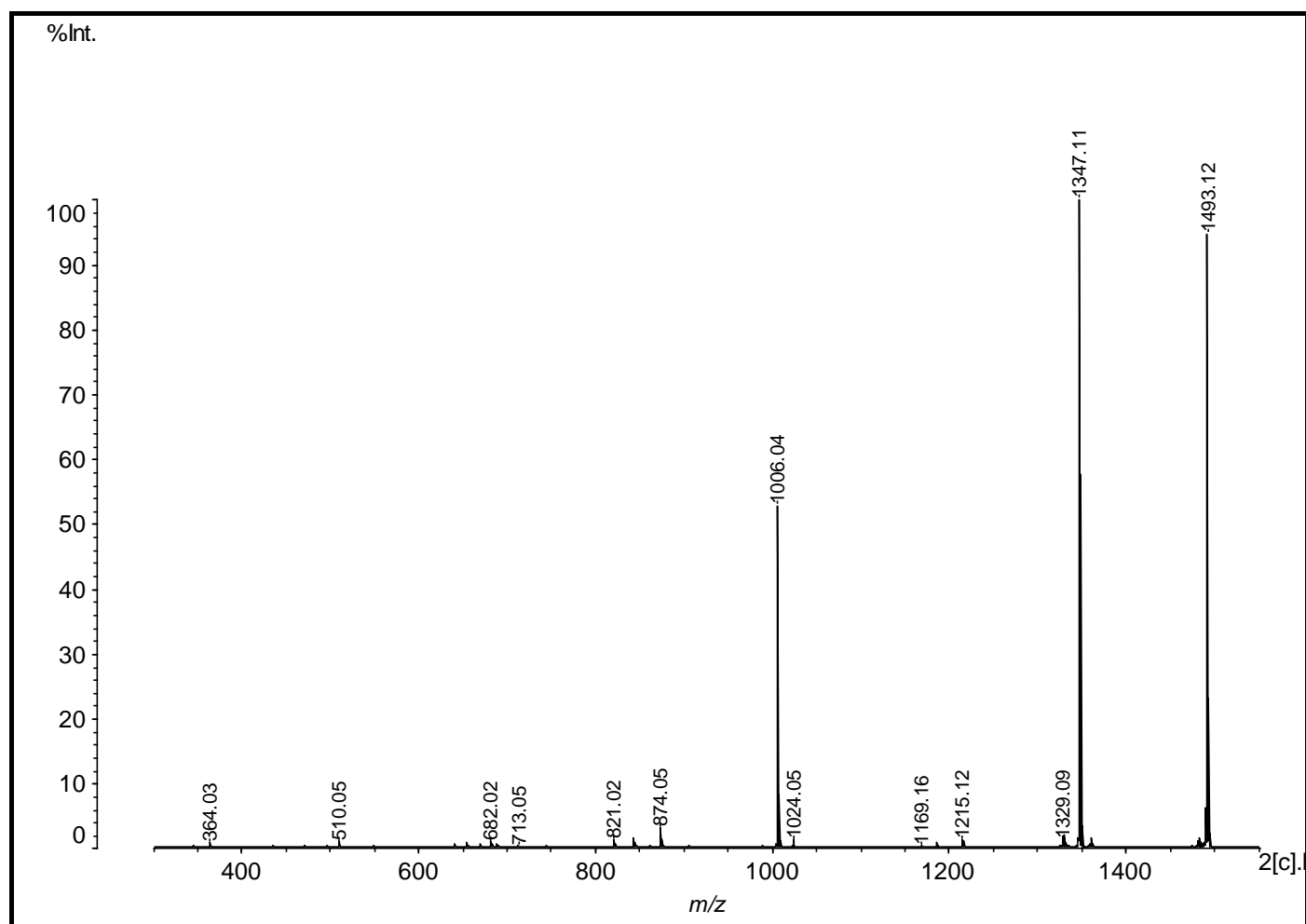

Supplement: S23 Fig — (A) MS spectrum, (B) MS/MS spectrum. (PDF) [file pone.0196800.s023.pdf]

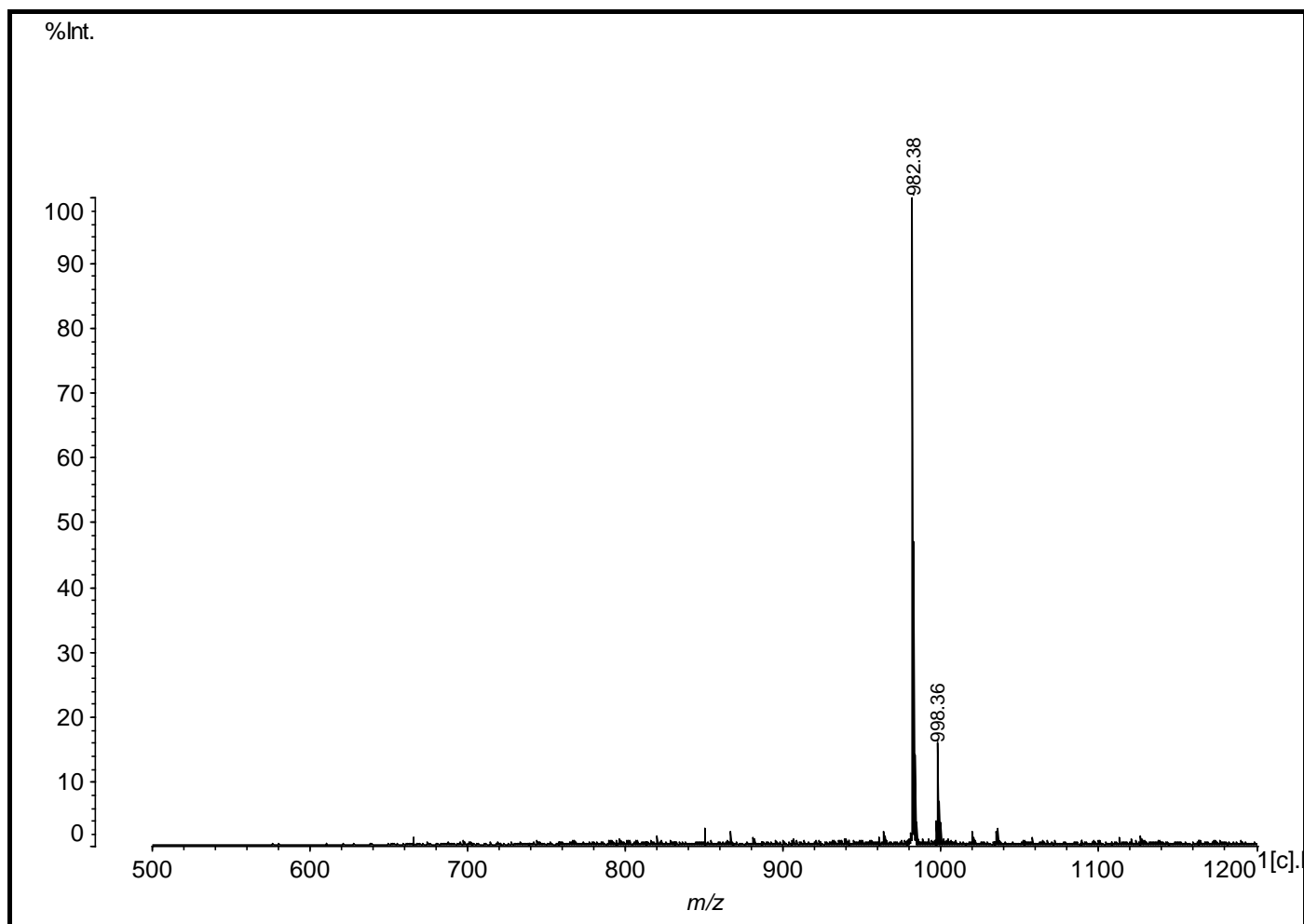

Supplement: S24 Fig — (PDF) [file pone.0196800.s024.pdf]

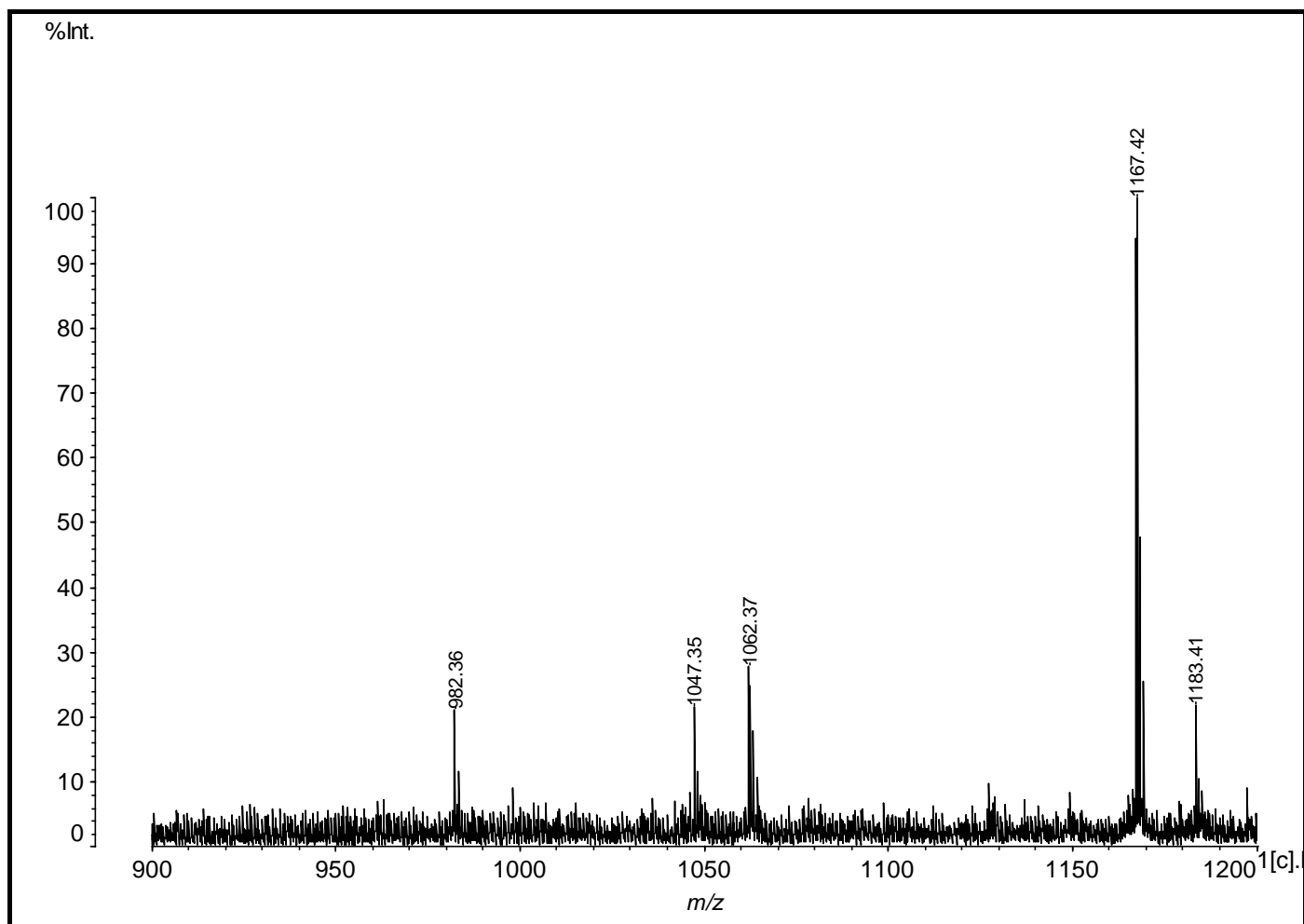

Supplement: S25 Fig — (PDF) [file pone.0196800.s025.pdf]

A

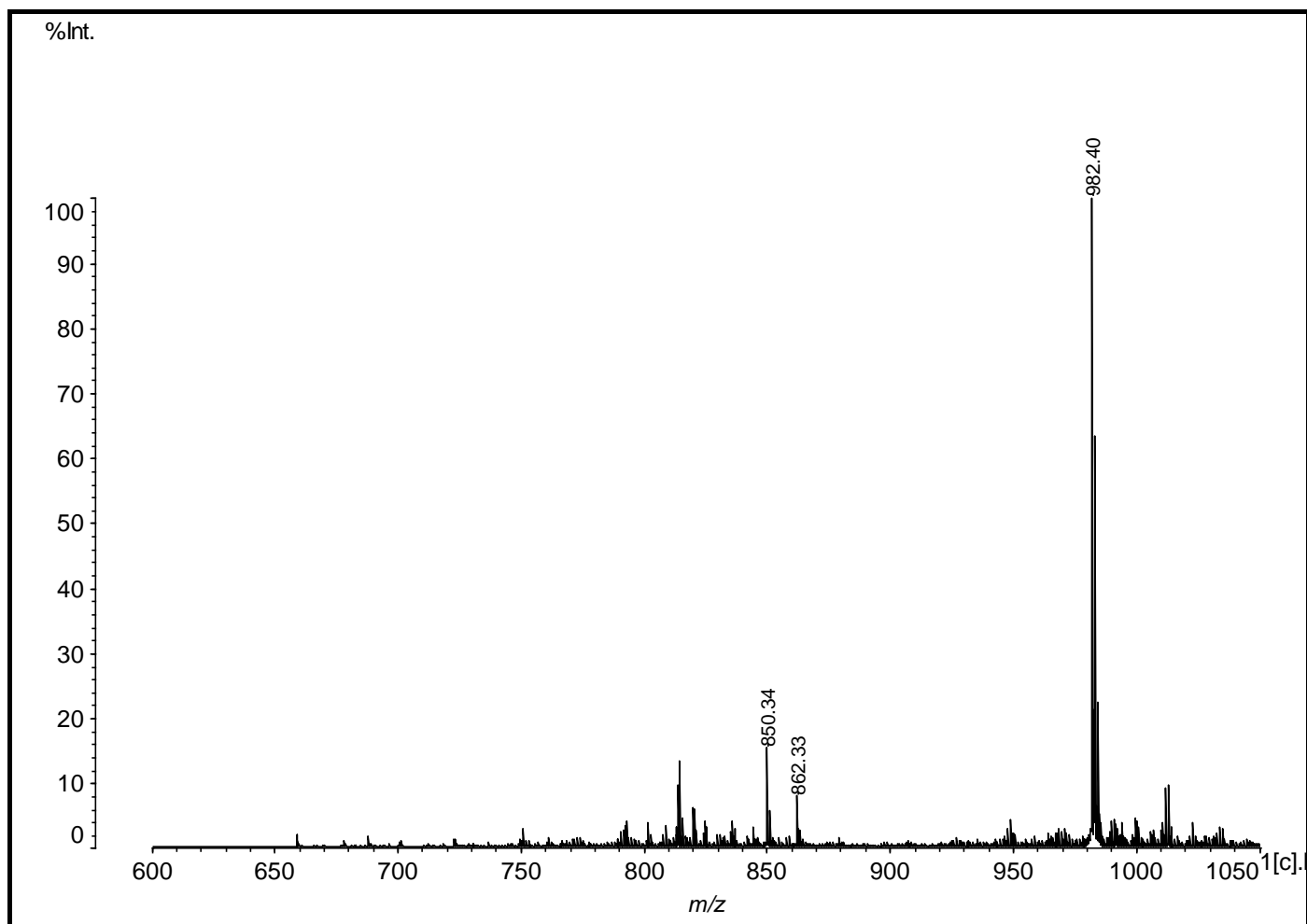

B

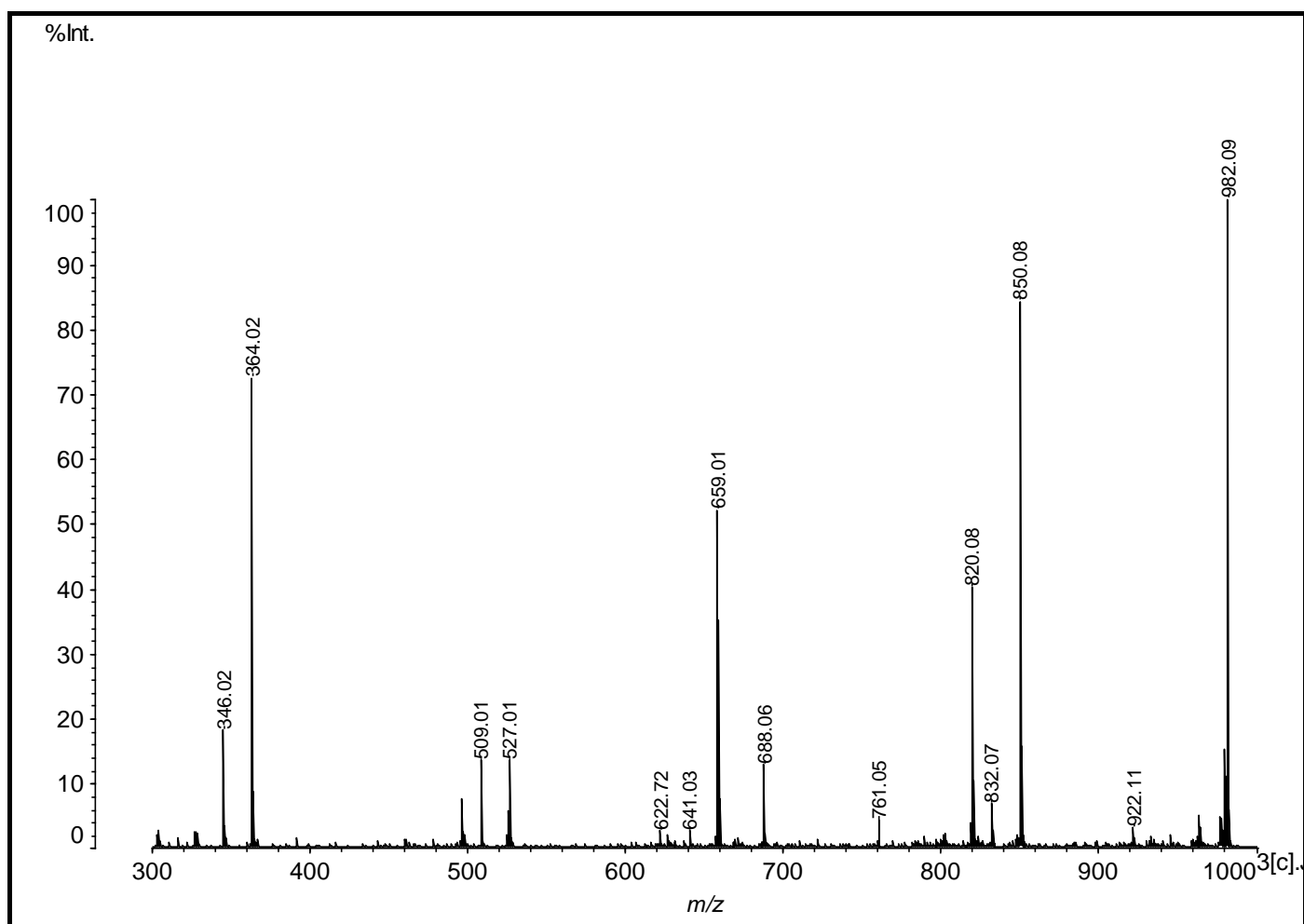

Supplement: S26 Fig — (A) MS spectrum, (B) MS/MS spectrum. (PDF) [file pone.0196800.s026.pdf]

A

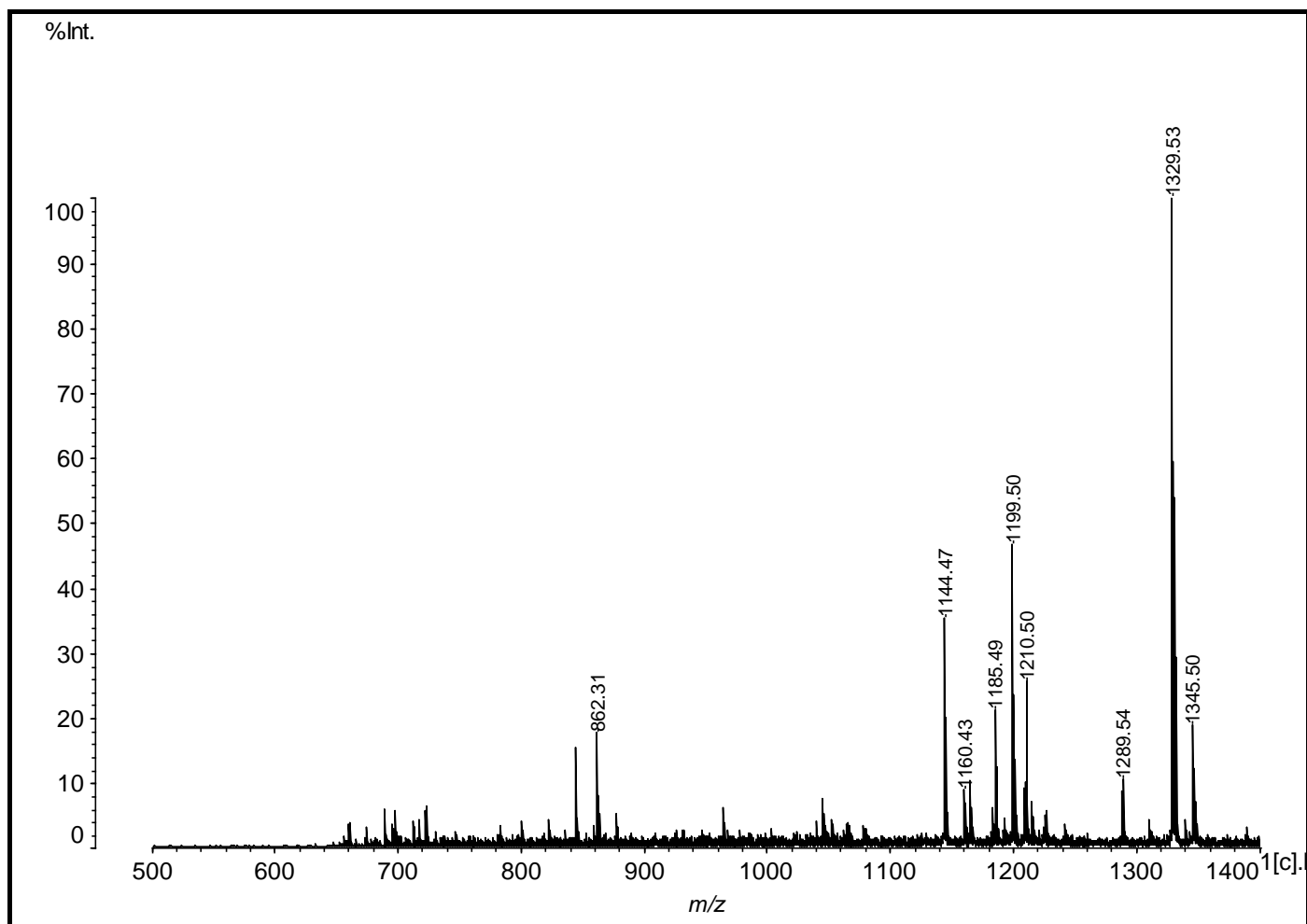

B

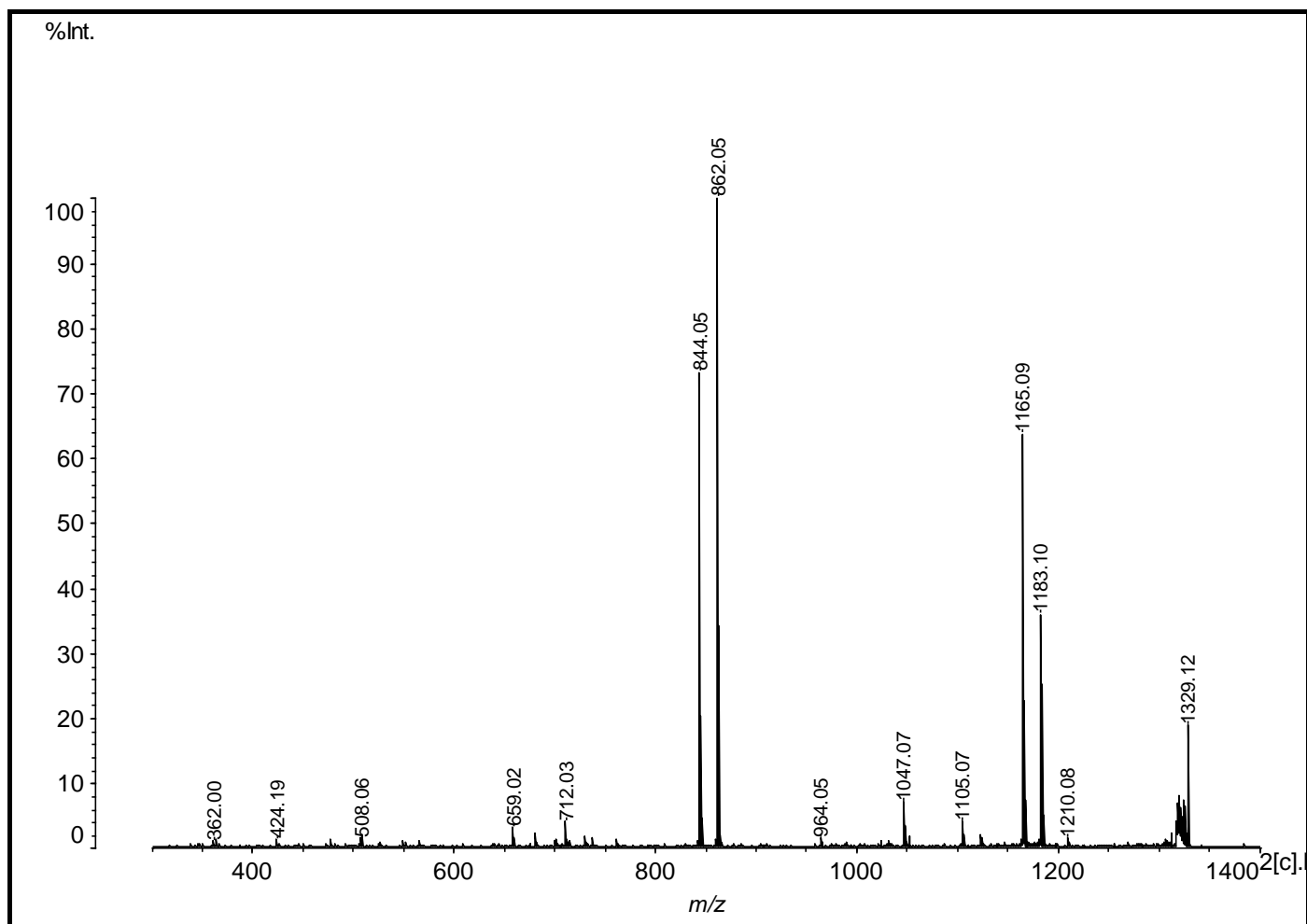

Supplement: S27 Fig — (A) MS spectrum, (B) MS/MS spectrum. (PDF) [file pone.0196800.s027.pdf]
